# Micronutrient Fortification of Commercially Available Biscuits Is Predicted to Have Minimal Impact on Prevalence of Inadequate Micronutrient Intakes: Modeling of National Dietary Data From Cameroon

**DOI:** 10.1093/cdn/nzaa132

**Published:** 2020-08-10

**Authors:** Demewoz Haile, Hanqi Luo, Stephen A Vosti, Kevin W Dodd, Charles D Arnold, Reina Engle-Stone

**Affiliations:** Department of Nutrition, University of California, Davis, CA, USA; Institute for Global Nutrition, University of California, Davis, CA, USA; Department of Nutrition, University of California, Davis, CA, USA; Institute for Global Nutrition, University of California, Davis, CA, USA; Department of Agricultural and Resource Economics, University of California, Davis, CA, USA; National Cancer Institute, National Institutes of Health, Bethesda, MD, USA; Institute for Global Nutrition, University of California, Davis, CA, USA; Department of Nutrition, University of California, Davis, CA, USA; Institute for Global Nutrition, University of California, Davis, CA, USA

**Keywords:** fortification, micronutrients, preschool children, women of reproductive age, dietary modeling, Cameroon

## Abstract

**Background:**

Voluntarily fortified snack products are increasingly available but are not necessarily formulated to meet known dietary nutrient gaps, so potential impacts on population micronutrient intake adequacy are uncertain.

**Objectives:**

We modeled the impacts of hypothetical micronutrient-fortified biscuits on inadequate micronutrient intake in children and women of reproductive age (WRA) in Cameroon.

**Methods:**

In a nationally representative survey stratified by macro-region (North, South, and Yaoundé/Douala), 24-h dietary recall data were collected from 883 children aged 12–59 mo and from 912 WRA. We estimated usual nutrient intake by the National Cancer Institute method for vitamin A, folate, vitamin B-12, zinc, and iron. We simulated the impact of biscuit fortification on prevalence of micronutrient intake below the estimated average requirement, given observed biscuit consumption, in the presence and absence of large-scale food fortification (LSFF) programs.

**Results:**

Biscuit consumption in the prior 24-h by children and WRA, respectively, ranged from 4.5% and 1.5% in the South, to 20.7% and 5.9% in Yaoundé/Douala. In the absence of LSFF programs, biscuits fortified with retinol (600 μg/100 g), folic acid (300 μg/100 g), and zinc (8 mg/100 g) were predicted to reduce the prevalence of inadequacy among children by 10.3 ± 4.4, 13.2 ± 4.2, and 12.0 ± 6.1 percentage points, respectively, in Yaoundé/Douala. However, when existing vitamin A–fortified oil, and folic acid–fortified and zinc-fortified wheat flour programs were considered, the additional impacts of fortified biscuits were reduced substantially. Micronutrient-fortified biscuits were predicted to have minimal impact on dietary inadequacy in WRA, with or without LSFF programs.

**Conclusions:**

Given observed patterns of biscuit consumption in Cameroon, biscuit fortification is unlikely to reduce dietary inadequacy of studied micronutrients, except possibly for selected nutrients in children in urban areas in the absence of LSFF programs. As voluntary fortification becomes increasingly common, modeling studies could help guide efforts to ensure that fortified products align with public health goals.

## Teaser

Fortification of commercially available biscuits with micronutrients has minimal impact to reduce prevalence of inadequate micronutrient intake in preschool children and women of reproductive age in Cameroon.

## Introduction

In low- and middle-income countries (LMIC), the prevalence of inadequate micronutrient intake and micronutrient deficiency is high, particularly in women and young children (1–4). Large-scale staple food fortification has been identified as an important strategy to address micronutrient malnutrition, and these food fortification programs have gained traction in a number of LMIC (5–7). However, large-scale staple food fortification might not successfully resolve inadequate intakes for several reasons: *1*) fortified foods might not reach the individuals at the highest risk of deficiency; *2*) staple foods might not provide sufficient amounts of micronutrients to meet the needs of vulnerable groups (e.g., to infants and young children) due to low consumption or low fortification concentrations; and *3*) fortification programs might not be implemented according to the target fortification concentrations (8, 9).

There is considerable interest within the private sector in voluntary fortification of their products with essential micronutrients (10, 11). Market-driven voluntary fortification programs could theoretically fill the gaps that are not addressed by large-scale food fortification (LSFF) programs, thus offering an opportunity for food manufacturers to contribute to combating the major global public health problem of micronutrient deficiencies (12). Voluntary fortification of foods such as breakfast cereals, beverages, milk, and confectionery products, has been shown to increase the intake and status of key micronutrients in many high-income countries (13–16). However, the potential impact of voluntary fortification of these products also depends on consumption patterns. For example, processed products might not be consumed by those most at risk of low intake if they are more expensive than nonindustrially produced alternatives. However, if these foods are fortified with nutrients that are already abundant in the diet, or if they are solely consumed by individuals with adequate intake, they can contribute to excessive micronutrient intakes (15). Additional concerns have also been raised about the overall nutritional quality of these products, specifically about the macronutrient profile and other dietary components (e.g., unhealthy lipid profile, salt, and added sugar), in the context of the global rise in obesity and noncommunicable diseases (11, 17).

Given these potential limitations, the likely impact of market-driven voluntary fortification programs on micronutrient intake should be examined and used to guide decisions about the influence of voluntary fortification programs. This study aimed to examine the potential impacts of voluntarily fortified processed foods on micronutrient intake using a case study of biscuit fortification in Cameroon. Biscuits are a popular food product amenable to voluntary fortification (18), and efficacy trials have shown that fortified biscuits improved micronutrient status of children in LMIC (19–23). We used data from a national sample of women and preschool children in Cameroon to model the potential impact on dietary micronutrient adequacy of fortifying biscuits that are already on the market (i.e., assuming that current consumption patterns do not change as result of introducing a fortified product). We examined this impact with and without a background of LSFF programs to fortify edible oil and wheat flour.

## Methods

### Study setting and data collection

We conducted a secondary analysis of dietary intake data from a national survey of Cameroon with a stratified multistage cluster design (24). The survey included participants from 3 geographic strata of Cameroon (North, South, and Yaoundé/Douala). The stratum of Yaoundé/Douala represented a mostly urban population (sampling from the 2 largest urban areas in Cameroon), whereas clusters drawn from the North and South represented a mixture of urban and rural locations. From each stratum, 30 clusters were randomly selected, and then 10 households (i.e., 10 women and 10 children) were selected per cluster.

Informed oral consent was obtained from the index woman for participation of herself and the index child. The study was approved by the Cameroon National Ethics Committee and the Institutional Review Board of the University of California, Davis (#200917294). Information on participants’ names as well as the cluster name was not entered into the electronic database, and birthdates were subsequently removed from the dietary dataset prior to analysis.

Data were collected on sociodemographic and economic status and individual characteristics such as child breast-feeding status. An interviewer-administered, multiple-pass 24-h dietary recall was used to collect dietary intake data from a total of 883 children aged 12–59 mo (with repeated assessments on a randomly selected subsample of 66 children) and 912 women of reproductive age (WRA; repeated on a randomly selected subsample of 72 women). Respondents were asked to report all foods and beverages consumed on the previous day. Food composition tables were constructed by using nutrient values from the Nutrition Coordinating Center Nutrient Database for Standard Reference (25), supplemented with values from the USDA, a food composition table from Uganda (26), the nutritional composition of commonly consumed dishes from Cameroon (27), and manufacturer information when necessary. For children who were reportedly still breast-fed at the time of the survey, we estimated the additional daily nutrient intake received from breast milk, as described below for each nutrient. Details of the dietary data collection and calculation of intakes of nutrients and fortifiable foods have been reported elsewhere (28).

In the context of this survey, “biscuit” refers to a variety of sweetened products that would be referred to as “cookies” in the United States; the most commonly reported brand was Parle G. Items recorded as *biscuit salé* (crackers) were not included. For this analysis, we estimated total biscuit consumption by summing the total weight in grams of biscuits consumed each day, regardless of brand or location of production (e.g., imported or domestic production). The modeled scenarios thus represent an optimistic situation with regard to the impact of voluntary biscuit fortification, which might in reality only be adopted by a subset of brands. The reach of biscuits as a delivery vehicle for micronutrients was defined as the proportion of individuals who consumed biscuits in the previous day. To assess the public health contribution of fortified biscuits, we calculated the prevalence of dietary inadequacy under different modeling scenarios, and defined effective coverage as the proportion of individuals who achieve adequate intake after fortified food is introduced. That is, effective coverage was calculated as the prevalence of dietary micronutrient inadequacy before biscuit fortification minus the prevalence of dietary inadequacy after biscuit fortification. We compared the mean intake of biscuits in consumers in the previous day by macroregion using 1-factor ANOVA.

### Dietary data modeling approach

We used the following approach to model the impact of fortified biscuits on the adequacy of nutrient intakes at the population level.

First, the baseline distribution of usual nutrient intakes for vitamin A, folate, vitamin B-12, zinc, and iron was estimated by using the “amount only” model of the National Cancer Institute (NCI) method (29, 30). We followed NCI recommendations to include covariates in the models. NCI recommends person-specific, time-dependent, and so-called “nuisance” factors to be included in the model as covariates. Including covariates helps to make the distribution of random effects more normally distributed and results in much greater precision (29, 31, 32). In this study, usual intake for preschool children was adjusted for age, sex, interviewer ID, sequence of interviews (i.e., first interview compared with subsequent interview), use of translator in the dietary interview, weekend (binary variable indicating weekend compared with weekday), breast-feeding status, maternal education (secondary/higher, primary, or no formal education), socioeconomic status [categorized into quintiles, as described previously (24)], and macroregion. For women, usual intake was adjusted for the following covariates: maternal age, interviewer ID, sequence of interviews, use of translator, weekend, maternal education (secondary/higher, primary, or no formal education), socioeconomic status, macroregion, and physiological status (nonpregnant/nonlactating or pregnant/lactating).

Estimated nutrient intake from breast milk was added to reported nutrient intake from other foods using the “shrink then add approach” (33). In the “shrink then add approach,” nutrient intakes from food sources are first processed through procedures to generate a representative sample of modeled usual intakes from food sources (i.e., the NCI amount only method), then the estimated usual nutrient intake from breast milk is added to each modeled intake to produce a representative sample of usual total nutrient intakes. Assumptions about the amount of each nutrient transferred through breast milk are described below.

We applied the estimated average requirement (EAR) cut-point method to estimate the prevalence of inadequate intake for vitamin A, folate, vitamin B-12, and zinc, whereas the full probability approach was applied for iron (34). We used age-specific EAR values of each nutrient for children, but for women the EAR values applied depend on physiological status (pregnant/lactating or nonpregnant/nonlactating). For these nutrients consumed nearly daily, we used the NCI “amount only” model. The prevalence of nutrient intake above the tolerable upper intake level (UL) was estimated for the highest fortification scenarios. The NCI “amount only” model was applied to estimate the prevalence of intakes above the UL for zinc and iron, but the NCI “two-part correlated model” was used for retinol and folic acid because these nutrients were episodically consumed. The NCI “two-part correlated model” jointly models the probability of consumption on a given day and the usual amount consumed on consumption days. Usual intake is the product of these 2 quantities, which can be correlated with one another (31, 32).

The fortification concentrations modeled were based on randomized trials in which increased concentrations of biological markers of micronutrient status were observed in participants fed fortified biscuits compared with control (22, 35–37) (**Table 1**). We also modeled fortification concentrations higher than those used in these studies under the assumption that such concentrations would also be technically feasible and would not cause adverse organoleptic profiles. We modeled the impact of biscuit fortification by adding the amount of target nutrient provided by each fortification scenario to the baseline nutrient intake (i.e., nutrient intake from all other dietary sources, excluding breast milk and fortified foods) for each recall. Then we estimated the population usual intake based on the NCI method, which involves estimating a distribution of “pseudo-persons” based on the observed intake on 1 or 2 days from different individuals (28, 38). For each nutrient, we examined the potential impact of fortified biscuits with or without the current LSFF programs. Because the predicted impacts from fortified biscuits were small, for simplicity we presented the results only for the highest biscuit fortification concentration; all other results are included in the supplemental material. We used the Fay modified balanced, repeated replication procedure to obtain appropriate SEs for the complex survey design. The prevalence of inadequate intake is presented with SE (percentage ± SE), unless otherwise noted. Additional nutrient-specific information on the dietary modeling approach is described in the following paragraphs.

**FIGURE 2:**
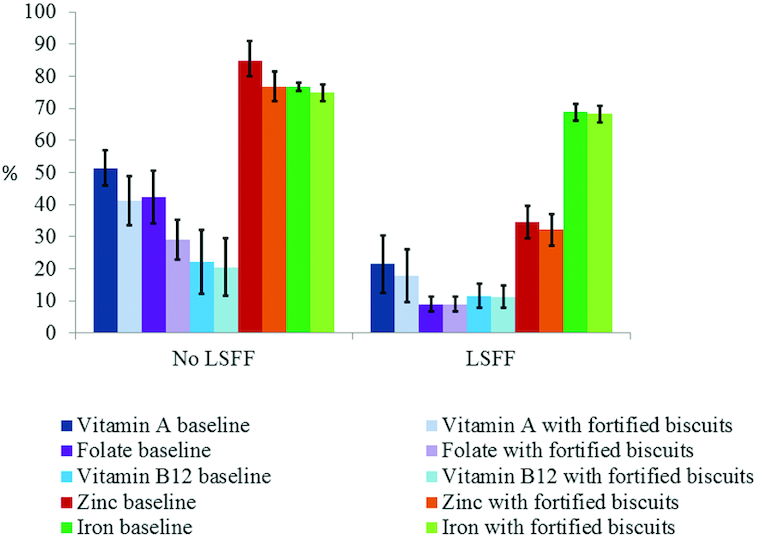
Prevalence of inadequate micronutrient intake among young children, with and without the presence of large-scale food fortification (LSFF) programs in Yaoundé/Douala. The bars represent proportion (± SE) below the estimated average requirement, except iron, for which prevalence of inadequate intake was estimated by using the full probability method. "No LSFF” refers to scenarios with micronutrient intake from natural food sources, in the absence of a LSFF program. ^“^LSFF” represents intakes in the presence of mandatory large-scale fortification of wheat flour with folic acid, vitamin B-12, zinc, and iron, and edible oil fortification with vitamin A. Modeled fortification concentrations per 100 g biscuits were: vitamin A: 600 µg retinol activity equivalents; folic acid: 300 µg; vitamin B-12: 2 µg; zinc: 8 mg; and iron: 15 mg. Effective coverage was calculated by subtracting the prevalence of inadequate intake at each fortification concentration from the baseline prevalence of inadequate intake.

**FIGURE 3:**
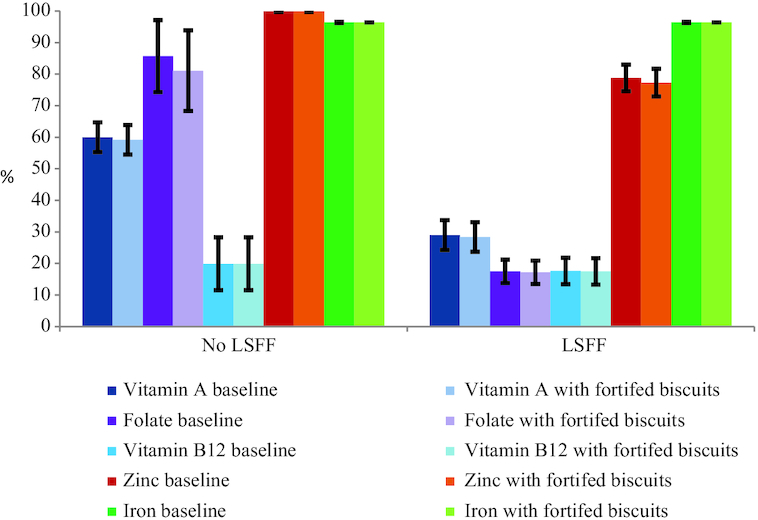
Prevalence of inadequate micronutrient intake among women of reproductive age, with and without the presence of large-scale food fortification (LSFF) programs in Yaoundé/Douala. The bars represent proportion (± SE) below the estimated average requirement, except iron, for which prevalence of inadequate intake was estimated by using the full probability method. "No LSFF” refers to scenarios with micronutrient intake from natural food sources, in the absence of a LSFF program. ^“^LSFF” represents intakes in the presence of mandatory large-scale fortification of wheat flour with folic acid, vitamin B-12, zinc, and iron, and edible oil fortification with vitamin A. Modeled fortification concentrations per 100 g biscuits were: vitamin A: 600 µg retinol activity equivalents; folic acid: 300 µg; vitamin B-12: 2 µg; zinc: 8 mg; and iron: 15 mg. Effective coverage was calculated by subtracting the prevalence of inadequate intake at each fortification concentration from the baseline prevalence of inadequate intake.

**TABLE 1:** Modeled fortification concentrations for selected nutrients added to biscuits, wheat flour, and refined vegetable oil

	Fortification concentration per 100 g biscuits (22, 35, 36)				Large-scale food fortification concentrations simulated (37)
Nutrient	Scenario 1	Scenario 2	Scenario 3	Scenario 4	
Vitamin A	200 µg RAE^1^	350 µg RAE	500 µg RAE	600 µg RAE	Oil fortification (12 mg/kg)
Folic acid	46 µg	80 µg	173 µg	300 µg	Wheat fortification (5 mg/kg)
Vitamin B-12	0.74 µg	0.98 µg	1.2 µg	2 µg	Wheat fortification (0.04 mg/kg)
Zinc	2 mg	4 mg	5 mg	8 mg	Wheat fortification (95 mg/kg)
Iron	5 mg	8 mg	11 mg	15 mg	Wheat fortification (60 mg/kg)

1 RAE, retinol activity equivalent.

#### Vitamin A

Dietary vitamin A was expressed as micrograms of retinol activity equivalents (RAEs). Region-specific estimates of vitamin A intake from breast milk were calculated based on the mean vitamin A content of the breast milk from each geographic stratum in the Cameroon national micronutrient survey (38) combined with estimates of average breast milk intake at 1–2 y of age (39) (**Supplemental Table 1**). The estimated ratio of within-person to between-person variance for vitamin A intake in women was extremely large, even after removing potential outliers as suggested by Davis et al. (31). To obtain reliable estimates in this case, we used a modified approach described in **Supplemental Methods**.

#### Folate

Dietary folate equivalents (DFEs) were calculated using the standard conversion methods (39). We assumed an average folate intake of 49 µg/d from breast milk for all children who were breast-fed (40).

#### Vitamin B-12

Region-specific vitamin B-12 intake from breast milk was estimated based on the mean vitamin B-12 content of the breast milk from each geographic stratum combined with estimates of average breast milk intake in children 1–2 y of age. The detailed calculation procedure for vitamin B-12 intake from breast milk is described in **Supplemental Table 2**. We estimated absorbable vitamin B-12 intake based on an absorption algorithm published by Doets et al. (41). We used the EAR cut-point method applied to “absorbed vitamin B-12” because the IOM EAR includes an assumption about the bioavailability of vitamin B-12 that might not be relevant to all foods. As a sensitivity analysis, we compared the prevalence of inadequate intake based on the total dietary vitamin B-12 intake and absorbable vitamin B-12.

#### Zinc

Total absorbable zinc intakes for young children (42) and women (43) were estimated by the absorption algorithms published by Miller and colleagues (43). As is well documented in the HarvestPlus 2012 consultative meeting (44), blanket assumptions of zinc bioavailability for all individuals cannot be justified any longer because equations are now available to estimate zinc absorption from the diet. Various estimates of physiological zinc requirement have been proposed by different expert groups, but estimates by the International Zinc Nutrition Consultative Group (IZiNCG) and IOM for adults were found to include errors resulting in underestimation and overestimation, respectively, of the physiological requirements. Thus, we chose to estimate the prevalence of inadequate intake for women based on the physiological requirement estimates of IZiNCG, which were corrected by Hambidge et al. (45). The physiological requirement for children was derived from that for adults. We assumed that the errors in estimation of the physiological requirement for adults carried over to young children. The error carried over from adult estimates of physiological requirements was considered most likely to affect the IZiNCG estimates for young children because the error was mostly in the estimation of the endogenous losses whereas the error in IOM estimates was in estimation of zinc loss through menstruation (45). Thus for young children, we used the IOM physiological requirements to estimate the prevalence of inadequate intake and effective coverage. However, as a sensitivity analysis, we presented the prevalence of inadequate intake estimated based on physiological requirement cutoff values of the IZiNCG and the European Food Safety Authority (EFSA) as well as based on total dietary zinc intake (EAR IZiNCG/IOM and EFSA) (46). Based on previous study (47), zinc intake estimated from breast milk for partially breast-fed infants aged 12–17 mo was 0.29 mg/d. We assumed that the bioavailability of zinc from breast milk is 50%, therefore zinc intake from breast milk is 0.145 mg/d.

#### Iron

Previous research showed that animal source foods are limited in the food supply in Cameroon (48), so for this study we assumed that only 10% of the total dietary iron intake is heme iron. Absorption of heme iron is estimated to be 25% (49) but the absorption of nonheme iron varies based on the consumption of dietary inhibitors (50). We used the algorithm of Armah et al. (50) to estimate the absorbable proportion of nonheme iron. Because we did not have data on vitamin C, meat, poultry, and fish, tea, and calcium, these variables were not included in the absorption algorithm. The regression coefficients for these variables are relatively small in the Armah et al. algorithm and thus this exclusion is unlikely to strongly influence the absorption adjustment (50). We combined estimated absorbable iron from heme and nonheme sources used as a total absorbable iron intake in the NCI model. The prevalence of inadequate iron intake was estimated by the full probability approach (34).

Given the low concentration of iron in mature breast milk (51, 52) and low prevalence of breast-feeding in this population, we did not include iron intake from breast milk in our modeling. We did a sensitivity analysis by including estimated iron from breast milk in total iron intakes (assuming concentrations of 0.2 mg iron/L, 0.549 L milk/d, and 50% absorption of breast milk iron); this addition had no substantial effect on the prevalence of inadequate intake or effective coverage.

## Results

### Reach of biscuits in Cameroon

**FIGURE 1:**
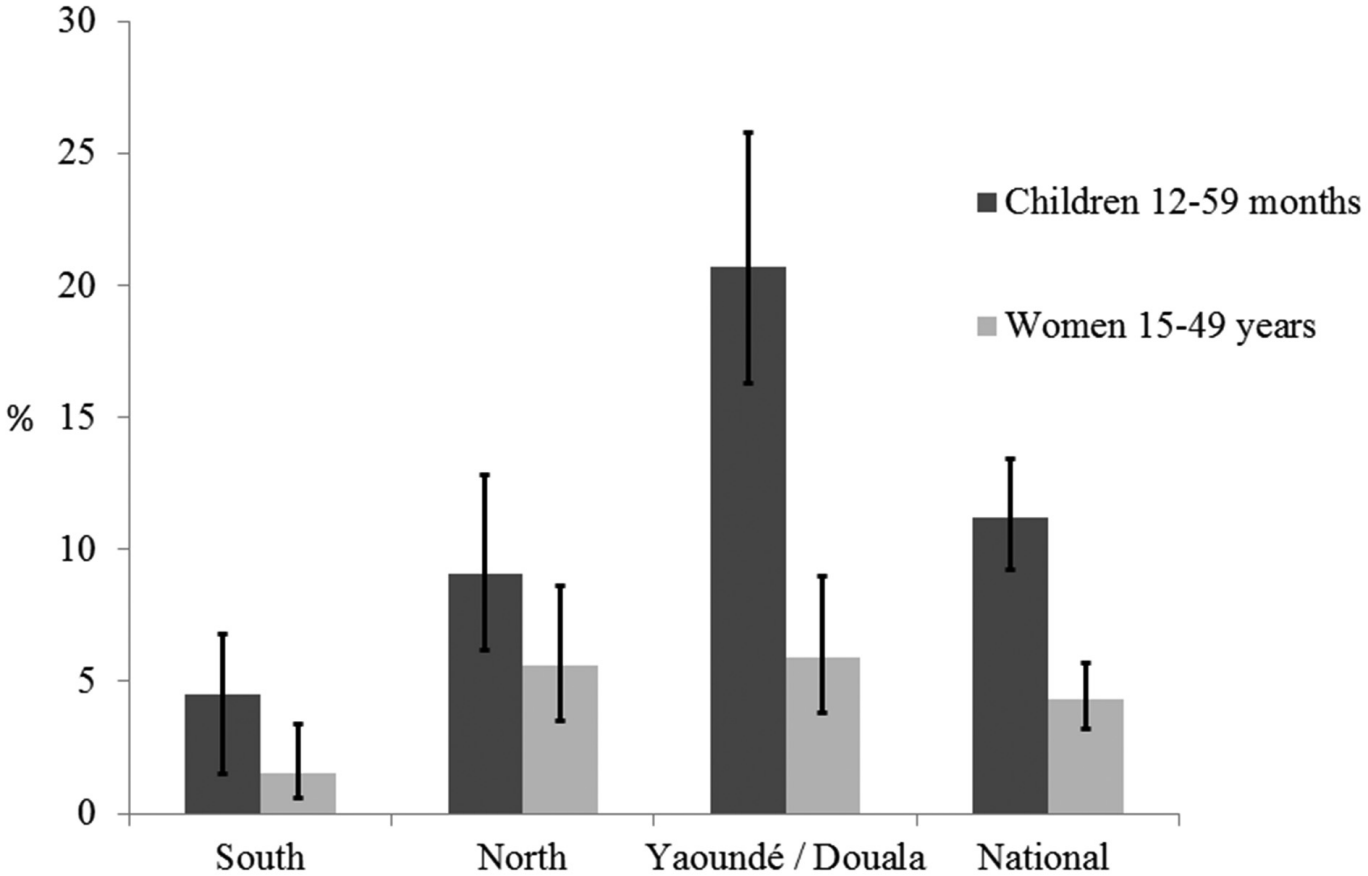
Reach of biscuits in the previous day in preschool children and women in Cameroon, nationally and by macroregion. Reach was defined as reported consumption of any type of biscuit on the previous day in 24-h dietary recall interviews. Error bars indicate 95% CI for reach of biscuits in the previous day.

There was regional variation in biscuit consumption, with the highest reach found in Yaoundé/Douala, where one-fifth of children consumed biscuits on the previous day (20.7%; 95% CI: 16.3, 25.8%) and the lowest reach observed in the South macroregion (4.5%; 95% CI: 2.3, 6.8%). Nationally, the reach of biscuits in preschool children was 11.2% (95% CI: 9.2, 13.4%) on the previous day (**Figure 1**), and the mean ± SE intake of biscuits in preschool children who consumed biscuits in the preceding day was 29.9 ± 2.7 g. The highest mean ± SE intake in biscuit consumers was in Yaoundé/Douala (36.4 ± 2.7 g) followed by the South macroregion (29.4 ± 5.3 g) and North macroregion (20.7 ± 2.6 g). The mean intake of biscuits in prior-day consumers was not significantly different between macroregions (*F*-value = 2.45; *P* value = 0.09).

Reach of biscuits on the previous day in WRA was low compared with preschool children: the highest reach was 5.9% (95% CI: 3.8, 9.0%) in Yaoundé/Douala (Figure 1). Nationally, the mean (± SE) intake of biscuits in women who consumed biscuits in the previous day was 51.6 ± 9.4 g (67.2 ± 20.9 g/d in Yaoundé/Douala, 51.8 ± 11.8 g/d in the North, and 23.0 ± 8.1 g/d in the South). The difference in mean intake of biscuit among prior-day consumers was not statistically significant by macroregions (*F*-value = 0.85; *P* value = 0.44).

### Nutrient intake and potential impact of fortified biscuits on inadequate nutrient intake of preschool children

#### Vitamin A

In children, the national median [25th percentile (P25), 75th percentile (P75)] usual vitamin A intake was 227 (102, 369) µg RAE/d in the absence of biscuit fortification (i.e., at the baseline) (**Table 2**; **Supplemental Table 3**). Vitamin A–fortified biscuits were predicted to have a greater impact on the prevalence of vitamin A inadequacy in Yaoundé/Douala compared with other macroregions in the absence of vitamin A–fortified oil. However, even in this scenario, the magnitude of change in prevalence of inadequate intake was modest: in Yaoundé/Douala the highest fortification concentration of 600 µg RAE/100 g biscuits would reduce the prevalence of vitamin A inadequacy from 51.4 ± 5.4% to 41.1 ± 7.7%. The effective coverage from fortified biscuits was <4 percentage points in South and North macroregions in the absence of vitamin A–fortified oil, and in all macroregions when vitamin A intake from fortified oil was included in the model (**Figure 2**; **Supplemental Table 4**).

**TABLE 2:** Median (25th, 75th percentiles) usual intake, assuming different concentrations of micronutrients added to biscuits, in the presence and absence of a large-scale fortification program in children aged 12–59 mo in Cameroon^1^

	Fortification concentration in biscuits^2^	In the absence of large-scale fortification			In the presence of large-scale fortification^3^		
Nutrient		South	North	Yaoundé/Doula	South	North	Yaoundé/Doula
Vitamin A, µg RAE/d							
	Baseline	320 (177, 581)	142 (69, 253)	186 (89, 383)	377 (225, 606)	233 (128, 303)	375 (208, 590)
	600 µg RAE	319 (183, 576)	156 (77, 261)	239 (120, 465)	382 (229, 612)	235 (135, 315)	417 (234, 634)
Folate, µg DFE/d							
	Baseline	128 (89, 177)	133 (92, 189)	129 (84, 183)	267 (165, 410)	245 (154, 374)	528 (321, 773)
	300 µg	131 (91, 183)	140 (96, 200)	158 (101, 225)	269 (165, 416)	250 (157, 384)	555 (335, 815)
Vitamin B-12, µg absorbable B-12/d							
	Baseline	0.7 (0.4, 1.1)	0.4 (0.2, 0.7)	0.7 (0.3, 1.3)	0.8 (0.4, 1.4)	0.5 (0.2, 1.0)	1.4 (0.7, 2.6)
	2 µg	0.7 (0.4, 1.3)	0.4 (0.2, 0.7)	0.7 (0.4, 1.4)	0.9 (0.4, 1.7)	0.5 (0.2, 1.0)	1.5 (0.7, 2.6)
Zinc, mg absorbable zinc/d							
	Baseline	0.8 (0.6, 1.0)	1.0 (0.8. 1.2)	0.8 (0.6, 1.0)	1.0 (0.7, 1.2)	1.1 (0.8. 1.3)	1.2 (0.9, 1.4)
	8 mg	0.8 (0.7, 1.0)	1.0 (0.8. 1.2)	0.9 (0.7, 1.0)	1.0 (0.7, 1.2)	1.1 (0.8. 1.3)	1.2 (0.9, 1.4)
Iron, mg absorbable iron/d							
	Baseline	0.3 (0.2, 0.6)	0.5 (0.3, 0.8)	0.3 (0.1, 0.5)	0.3 (0.2, 0.5)	0.4 (0.3, 0.6)	0.3 (0.1, 0.5)
	15 mg	0.3(0.2, 0.6)	0.5 (0.3, 0.8)	0.3 (0.1, 0.6)	0.4 (0.2, 0.6)	0.5 (0.3, 0.7)	0.5 (0.3, 0.7)

1 Median (25th, 75th percentiles) usual intake included estimated micronutrient intake from breast milk for children who were breast-fed at the time of the survey. Absorbable vitamin B-12, absorbable zinc, and absorbable iron were estimated using published algorithms, as described in detail in the text (41, 42, 50). DFE, dietary folate equivalent; RAE, retinol activity equivalent.

2 Nutrient fortification concentration is per 100 g biscuits. Baseline indicates total nutrient intakes without added micronutrients in biscuits.

3 Large-scale fortification concentrations: wheat flour fortification with folic acid (5 mg/kg), vitamin B-12 (0.04 mg/kg), zinc (95 mg/kg), iron (60 mg/kg), and edible oil fortification with vitamin A (12 mg/kg).

#### Folate

At baseline, the prevalence of inadequate folate intake was >40% in all macroregions. The simulation modeling showed that fortifying biscuit with 300 µg folic acid/100 g biscuits was predicted to increase the median usual intake to 158 (101, 225) µg DFE/d in Yaoundé/Douala (Table 2). This translates to a reduction in the prevalence of inadequate folate intake of 13 percentage points (Figure 2); however, the impact of folic acid–fortified biscuits on inadequate folate intake was almost zero when folate intake from fortified wheat flour was included in the model (Supplemental Table 4).

#### Vitamin B-12

The baseline national median (P25, P75) usual absorbable intake of vitamin B-12 was 0.6 (0.3, 1.1) µg/d. The maximum effective coverage achieved from the highest fortification concentration of vitamin B-12–fortified biscuits (2 µg/100 g) was <2 percentage points without vitamin B-12–fortified wheat flour in the model and <1 percentage point with fortified wheat flour in the model (Figure 2; Supplemental Table 4). Our sensitivity analysis found that the prevalence of inadequate intake estimated based on total dietary vitamin B-12 intake was somewhat lower than the prevalence of inadequate intake estimated based on absorbable vitamin B-12 (∼24% compared with ∼31% inadequate intake nationally), but the estimated effective coverage (i.e., impact of the candidate intervention) was comparable at <1 percentage point (**Supplemental Table 5**).

#### Zinc

The national baseline median (P25, P75) usual absorbable daily zinc intake was 0.9 (0.7, 1.1) mg. The maximum effective coverage from the highest fortification concentration of biscuits with zinc (8 mg /100 g) was ∼12 percentage points (based on IOM physiological requirement) in Yaoundé/Douala, but this dropped to 2 percentage points when zinc-fortified flour was included in the model (Supplemental Table 4**)**. As presented in **Supplemental Table 6**, the prevalence of zinc inadequate intake varied substantially depending on the cutoff values applied. The national prevalence of zinc inadequacy was 6.6 ± 4.7% based on IZiNCG physiological requirement cutoffs, but 78.4 ± 3.7% of preschool children had inadequate zinc intake based on EFSA physiological requirement cutoffs.

#### Iron

The national baseline median (P25, P75) usual absorbable daily iron intake was 0.4 (0.2, 0.6) mg. The potential impact of iron-fortified biscuits was either very small (<2 percentage points) or zero in all macroregions of Cameroon in the absence or presence of presence of iron-fortified wheat flour in the model (Figure 2; Supplemental Table 4).

### Nutrient intake and impact of fortified biscuits on inadequate intake among WRA

#### Vitamin A

In women, the national baseline median (P25, P75) usual vitamin A intake was 536 (262, 765) µg RAE/d and increased to 653 (400, 893) µg RAE/d when vitamin A intake from fortified oil was included in the model. Vitamin A–fortified biscuits at a fortification concentration of 600 µg RAE/100 g were predicted to increase the median usual intake of vitamin A from 531 (436, 622) to 578 (475, 647) µg RAE/d in Yaoundé/Douala (**Table 3**; **Supplemental Table 7**). The effective coverage from vitamin A–fortified biscuits was <1 percentage point in all macroregions with the presence or absence of vitamin A–fortified oil in the model (**Figure 3**; **Supplemental Table 8**).

**TABLE 3:** Median (25th, 75th percentiles) usual intake, assuming different concentrations of micronutrients added to biscuits, in the presence and absence of large-scale fortification program in women aged 15–49 y in Cameroon^1^

	Fortification concentration in biscuits^2^	In the absence of large-scale fortification			In the presence of large-scale fortification^3^		
Nutrient		South	North	Yaoundé/Doula	South	North	Yaoundé/Doula
Vitamin A, µg RAE/d							
	Baseline	769 (642, 888)	230 (169, 304)	531 (436, 622)	772 (602, 952)	369 (272, 481)	871 (676, 1064)
	600 µg	789 (675, 883)	250 (178, 323.)	578 (475, 647)	777 (604, 961)	381 (278, 500)	892 (689, 1095)
Folate, µg DFE/d							
	Baseline	279 (219, 344)	351 (279, 430)	255 (200, 310)	441 (292, 627)	556 (385, 771)	761 (500, 1062)
	300 µg	288 (227, 348)	365 (294, 443)	275 (219, 326)	445 (296, 632)	567 (393, 786)	778 (512, 1086)
Vitamin B-12, µg absorbable B-12/d							
	Baseline	3.9 (2.0, 6.7)	2.4 (2.2, 4.4)	5.0 (2.7, 8.0)	3.9 (2.0, 6.9)	2.4 (1.2, 4.4)	7.4 (3.5, 12.8)
	2 µg	4.0 (2.2, 6.7)	2.6 (1.4, 4.5)	4.9 (2.5, 8.2)	4.4 (2.1, 8.2)	3.0 (1.4, 5.7)	7.4 (3.5, 12.9)
Zinc, mg absorbable zinc/d							
	Baseline	1.2 (0.9, 1.4)	2.2 (1.8, 2.7)	1.2 (0.9, 1.5)	1.5 (1.1, 2.0)	2.7 (2.1, 3.4)	2.3 (1.6, 2.9)
	8 mg	1.2 (0.9, 1.4)	2.2 (1.8, 2.7)	1.2 (0.9, 1.5)	1.5 (1.1, 2.0)	2.7 (2.1, 3.5)	2.3 (1.7, 3.0)
Iron, mg absorbable iron/d							
	Baseline	0.5 (0.3, 0.7)	0.7 (0.5, 1.0)	0.5 (0.3, 0.8)	0.5 (0.3, 0.7)	0.7 (0.5, 0.9)	0.4 (0.2, 0.6)
	15 mg	0.5 (0.3, 0.7)	0.7 (0.5, 1.0)	0.5 (0.3, 0.8)	0.5 (0.3, 0.7)	0.7 (0.5, 1.0)	0.6 (0.3, 0.8)

1 Median (25th, 75th percentiles) usual intake. Absorbable vitamin B-12, absorbable zinc, and absorbable iron were estimated using published algorithms, as described in detail in the text (41, 43, 50). DFE, dietary folate equivalent; RAE, retinol activity equivalent.

2 Nutrient fortification concentration is per 100 g biscuits. Baseline indicates total nutrient intakes without added micronutrients in biscuits.

3 Large-scale fortification concentrations: wheat flour fortification with folic acid (5 mg/kg), vitamin B-12 (0.04 mg/kg), zinc (95 mg/kg), iron (60 mg/kg), and edible oil fortification with vitamin A (12 mg/kg).

#### Folate

The national baseline median (P25, P75) usual intake increased from 293 (229, 367) µg DFE/d to 529 (343, 768) µg DFE/d when folic acid–fortified wheat flour was included in the model. The impact of folic acid–fortified biscuits on prevalence of inadequacy was small (<5%) in all macroregions of Cameroon in the absence of folic acid–fortified flour in the model. With folic acid–fortified flour included in the model, the effective coverage of folic acid–fortified biscuits was <1 percentage point in all macroregions (Figure 3; Supplemental Table 8).

#### Vitamin B-12

The national baseline median (P25, P75) usual absorbable daily vitamin B-12 intake was 3.4 (1.6, 6.3) µg. In WRA, the maximum effective coverage of fortified vitamin B-12 biscuits was predicted to be 5 percentage points in all macroregions of Cameroon, regardless of the presence or absence of vitamin B-12–fortified flour (Figure 3; Supplemental Table 8).

#### Zinc

The national baseline median (P25, P75) usual absorbable daily zinc intake was 1.4 (1.0, 1.9) mg, and this increased to 2.0 (1.4, 2.8) mg when zinc-fortified wheat was included in the model. Zinc-fortified biscuits were predicted to have no effect on the inadequacy of zinc intake in women in all macroregions in the presence or absence of zinc-fortified wheat flour (Figure 3; Supplemental Table 8).

#### Iron

The national baseline median (P25, P75) usual absorbable daily iron intake was 0.6 (0.4, 0.8) mg. The median (P25, P75) usual absorbable iron intake per each fortification level of biscuits in the presence and absence of a wheat fortification program is shown in Table 3 and Supplemental Table 7. Iron-fortified biscuits had no impact on inadequate iron intake of WRA in either of the scenarios, that is, in the presence or absence of iron-fortified flour (Figure 3; Supplemently Table 8).

### Impact of fortified biscuits on prevalence of micronutrient intake above the UL in preschool children and WRA

The prevalence of retinol intake above the UL was almost zero (<0.5%) for any fortification level of biscuits in the presence or absence of an oil fortification program in all macroregions of Cameroon for both preschool children and WRA. Similarly, folic acid– and iron-fortified biscuits were predicted to have no effect on the prevalence of folic acid or total iron intakes above the UL in the presence or absence of fortified wheat flour. Total zinc intake above the UL was not observed for women in any modeled scenarios; however, for preschool children, the inclusion of fortified biscuits in the model increased the prevalence of total zinc intakes above the UL to ∼1–4%, depending on the macroregion, with or without fortified wheat flour in the model.

## Discussion

Given the increasing presence of packaged snack foods in markets globally (17, 53) and the interest in public-private partnerships for reducing malnutrition, we conducted this modeling study to examine the potential impacts of fortified biscuits on adequacy of micronutrient intakes at the population level in Cameroon. As a fortification vehicle, the reach and amount consumed of biscuits were limited when this survey was conducted in 2009: nationally, biscuits were consumed by 4% of women and 11% of children on the previous day, although reach tended to be greater among young children in urban macroregions (in Yaoundé/Doula, 20% consumed biscuits on the previous day). Previous studies showed consumption of biscuits is relatively higher in urban settings compared with rural settings, which might be related to better availability of biscuits in urban settings (54). In the absence of a well-performing LSFF program, our modeling suggested that biscuit fortification could contribute modestly to adequacy of intakes of vitamin A, folate, and zinc in children in Yaoundé/Douala (∼10–13 percentage points decrease in inadequate intakes), but not for vitamin B-12 and iron. In addition, for women, micronutrient-fortified biscuits are likely to have no impact on micronutrient inadequacy of intakes for all target nutrients at the modeled fortification levels in the absence or presence of LSFF programs. Although it is possible that providing small amounts of additional micronutrients could be beneficial, even if the amounts are insufficient to meet the EAR, the low reach of biscuits among the groups at risk of deficiency is a major limitation for addressing inadequate micronutrient intake.

We defined reach as consumption of biscuits on the previous day, so it should be noted that use of a longer recall period would increase the observed proportion of the population that consumes biscuits. Qualitative food frequency data from this same study indicated that 69% of children reportedly consumed biscuits in the previous week, with a mean frequency of 4 times per week (24, 55). Although this number might represent an overestimate due to use of an aggregate food category for biscuits and greater potential for recall bias in an FFQ, it is likely that a greater proportion of children would have reported biscuit consumption when a longer time frame is considered compared with the 24-h recall. Although differences in frequency of intake between individuals would have important implications for the effects on those individuals, the modeling methods applied here to the 24-h recall data represent the effect on population-level dietary adequacy on any given day.

If mandated programs for large-scale staple food fortification function well, our simulations predict little to no additional impact of biscuit fortification for any nutrient included in this modeling study (vitamin A, folate, vitamin B-12, zinc, and iron). This likely reflects the fact that consumption patterns of biscuits are similar to consumption patterns of oil and wheat flour: reach of oil and wheat flour was greatest in the Yaoundé/Doula macroregion (24). Thus, if the oil and wheat flour programs are working well, voluntary fortification of biscuits with vitamin A, folate, and zinc would be redundant with these LSFF programs and provide limited additional benefit at the population level. Theoretically, overlap in consumption of multiple sources of fortified foods could put some groups at risk of exceeding the UL if program design (e.g., selection of fortification levels) does not account for this overlap. In practice it is challenging to adapt the design of public health programs to take into account these changes to the food system because often there is no mechanism for tracking the composition of voluntarily fortified products (56). In these scenarios with combined LSFF and fortified biscuits, we did not see evidence that biscuit fortification would contribute to intakes above the UL at the modeled fortification levels. The 1 exception was for total zinc intake, for which there was a slight increase in prevalence of zinc intake above the UL in preschool children after zinc-fortified biscuits were included in the model. Zinc intakes above the UL have been commonly observed in children, even in the absence of zinc interventions programs, and are unlikely to be harmful (57). Several zinc supplementation trials used zinc supplementation doses higher than the current UL and did not report serious adverse effects (58–60). Thus, our results do not suggest any harm from high micronutrient intakes as a result of biscuit fortification.

In light of observed biscuit consumption patterns, greater reach of biscuits, and possibly greater fortification levels or amounts consumed, would be required to increase the effective coverage of fortified biscuits in Cameroon. However, promoting increased consumption of biscuits would not be desirable from the point of view of improving overall dietary quality. The rate of increase in consumption of processed foods that are high in salt, (unhealthy) fat, and sugar is already the fastest in history (61), and the prevalence of overweight in both adults and children continues to increase (62). Studies have reported a statistically significant association of processed snack food consumption with low nutrient adequacy, micronutrient deficiencies, and lower height-for-age *z*-score in LMIC (63, 64). Food industries can use micronutrient fortification as a marketing strategy to claim that their products are healthy (65). Although addition of micronutrients might help to address 1 aspect of malnutrition (micronutrient deficiencies), there is evidence that fortification claims on snack foods can be associated with selection of products that are less healthy overall. In 1 study, consumers exposed to fortification claims were *1*) less likely to look for nutrition information on the label of a fortified product, *2*) more likely to select the fortified product for purchase, and *3*) more likely to perceive the fortified product as healthier (66). One of the pillars of WHO's response to the double burden of malnutrition is to ensure that nutrition interventions are consistent with the “do no harm” framework (67). The *Lancet* series on the double burden of malnutrition proposed double-duty actions for nutrition, which include avoiding the consumption of foods, snacks, and beverages high in energy, sugar, fat, and salt, particularly for children (68). Consideration of fortified biscuits as a potential strategy for increasing micronutrient intakes requires attention to the unintended consequences for other forms of malnutrition.

This study has its strengths and limitations. This study uses nationally representative data and rigorous modeling methods to simulate the potential impact of voluntary processed foods for addressing micronutrient deficiencies in an African setting. This type of simulation modeling can be used in combination with cost data, an approach that is useful to both the private sector and nutrition program advocates to understand the cost-effectiveness of voluntary fortification of snack foods alone and in comparison with other strategies to reduce micronutrient deficiencies.

However, the findings of this study should be interpreted with caution. This analysis applied to untargeted fortification of snack foods, assuming no changes in consumption patterns. The results do not apply to specially formulated fortified products targeted to groups at risk of micronutrient deficiency, such as fortified complementary foods. The data might not reflect the current scenario because the survey was conducted 10 y ago and global trends suggest that consumption of processed foods has increased over time, which would lead our analysis to underestimate the effects of fortified biscuits on micronutrient intakes. However, because we combined all brands and types of biscuits, the results could potentially overestimate the contribution to micronutrient intakes, compared with a scenario where only a few brands participate in voluntary fortification.

We used the change in prevalence of inadequate intakes, that is, effective coverage, as our primary measure of benefits. This approach ignores the potential benefits of providing additional micronutrients in amounts that are not large enough to reach the EAR because there is no obvious way to quantify the “dose–response” relation between small increases in micronutrient intake and micronutrient status or health outcomes. This would tend to underestimate the benefits of biscuit fortification, especially among individuals with severe micronutrient deficiencies.

In this analysis, biscuits were selected as 1 example of a processed snack food, so this study might not be generalizable to other processed foods. However, it is generally true that any product with low reach would have limited impact on dietary adequacy at the population level. Though there are studies that showed an elevated prevalence of obesity and overweight in Cameroon (69, 70), this study did not model the potential effects of processed foods, including biscuits, on risk of noncommunicable diseases. This is an important concern, which emphasizes the need to explore other strategies to increase dietary micronutrient adequacy, including access to nutrient-dense foods such as animal products, fruits, or vegetables.

The wide differences in the estimated physiological requirement of children for zinc between IZiNCG, IOM, and EFSA pose a challenge to determine the effect of nutrition interventions, including fortified biscuits, on dietary zinc adequacy. The benefit estimated from dietary zinc intervention varies depending on the cutoff value used, which might lead to a wrong conclusion with implications for policy decisions. It is also important to note that the absorption algorithms applied to estimate absorbable nutrient intake (vitamin B-12, zinc, and iron) have their own limitations and they should be taken into consideration when interpreting our findings. Nevertheless, the conclusion regarding the low impact of biscuit fortification on zinc intake is robust to the modeling method and cutoff used.

In conclusion, biscuit consumption in Cameroon was relatively uncommon, except among children in Yaoundé/Douala. However, inadequate micronutrient intakes were generally less common and the reach of large-scale staple food fortification programs was generally greater, in these same areas. As a result, biscuit fortification is unlikely to reduce the prevalence of inadequate micronutrient intakes in this setting. Fortified biscuits might contribute modestly to nutrient adequacy in the absence of large-scale micronutrient fortification programs, particularly in urban areas, for vitamin A, folate, and zinc in children, but this contribution must be weighed against other nutrition priorities. As processed snack foods with added micronutrients appear more frequently in markets throughout the world, modeling studies are useful to assess the extent to which these products will align with and contribute to public health goals.

## Supplementary Material

nzaa132_Supplemental_File

## Notes

The dietary data collection was supported by the Michael and Susan Dell Foundation. The preliminary analyses of dietary intake data and the development of analytical techniques were supported in part by a grant to UC Davis by the Bill & Melinda Gates Foundation (grant #OPP1170661). Partial support for this research was provided by a training gift to UC Davis from Mars Inc. The funding agencies had no role in the study design, analysis, or interpretation.

Author disclosures: The authors report no conflicts of interest.

Supplemental Methods and Supplemental Tables 1–8 are available from the “Supplementary data” link in the online posting of the article and from the same link in the online table of contents at https://academic.oup.com/cdn.

Abbreviations used: DFE, dietary folate equivalent; EAR, estimated average requirement; EFSA, European Food Safety Authority; IOM, Institute of Medicine; IZiNCG, International Zinc Nutrition Consultative Group; LMIC, low- and middle-income countries; LSFF, large-scale food fortification; NCI, National Cancer Institute; P25, 25th percentile; P75, 75th percentile; RAE, retinol activity equivalent; UL, upper limit; WRA, women of reproductive age.
