# Supplementary material for: Micronutrient Fortification of Commercially Available Biscuits Is Predicted to Have Minimal Impact on Prevalence of Inadequate Micronutrient Intakes: Modeling of National Dietary Data From Cameroon
Source: Curr Dev Nutr. 2020 Aug 10;4(9):nzaa132. doi: 10.1093/cdn/nzaa132 (PMC7467246; doi:10.1093/cdn/nzaa132)
Supplement: nzaa132_Supplemental_File [file nzaa132_supplemental_file.docx]

**Micronutrient fortification of commercially available biscuits is predicted to have minimal impact on prevalence of inadequate micronutrient intakes: modeling of national dietary data from Cameroon. Demewoz Haile. Online Supplementary Material**

**Online Supplementary Material** includes modified vitamin A usual intake estimation method for women, vitamin A breast milk concentration, vitamin B12 breast milk concentration, usual nutrient intake distribution, prevalence of inadequate intake and references for the supplementary material

1. Supplementary Table 1 : Vitamin A breast milk concentration
2. Supplementary Table 2 : Vitamin B12 breast milk concentration
3. Supplementary Table 3 : Usual nutrient intake distribution among children
4. Supplementary Table 4 : Predicting the impact of fortified biscuits, assuming different level of biscuit fortification, on prevalence of inadequate intake among children in the absence and presence of large scale fortification programs
5. Supplementary Table 5: Sensitivity analysis to compare the prevalence of inadequacy of vitamin B12 for preschool children estimated based on total dietary vitamin B12 intake and absorbable vitamin B12 at different level of biscuits fortification
6. Supplementary Table 6: Sensitivity analysis to compare the prevalence of inadequate zinc intake based on cutoff values recommended by different expert groups
7. Supplementary Table 7: Usual nutrient intake distribution among women
8. Supplementary Table 8: Predicting the impact of fortified biscuits, assuming different level of biscuit fortification, on prevalence of inadequate intake among women in reproductive age in the absence and presence of large scale fortification programs

**Supplementary methods**

**Modified usual intake modeling of Vitamin A for women**

Note that this procedure was adopted in a specific case where the standard NCI method produced unreliable estimates due to special features of the data set under study. Therefore, replicating this procedure for other studies is not recommended as part of a primary analysis. The interpretation of the finding from this study should consider this limitation.

The extremely high ratio of within- to between-person variance in Vitamin A intake among women seemed to be caused by extreme differences between pairs of observations in the subsample with two recalls. This caused the one-part NCI model to produce estimated usual intake distributions that consisted of spikes at the mean of each covariate pattern, because the between-person variance remaining in the presence of covariates was estimated as essentially zero.

To get more realistic estimates, we categorized each 24HR as either a “low-intake day” or “high-intake day” according to whether or not the reported Vitamin A on the day was less than or equal to the EAR(500 µg RAE) of the distribution of 24HRs. We created a working data set of low intakes by replacing high intakes with zeros, then applied the two-part correlated NCI model to the working data set to generate a representative sample of individual probabilities (*p_i_*_,_*_low_*) to have a low-intake day and individual usual intakes (*A_i_*_,_*_low_*) of Vitamin A on low-intake days. Individual probabilities to have a high-intake day were computed as *p_i_*_,_*_high_* = (1 - *p_i_*_,_*_low_*). This part of the modeling accounted for all covariates under consideration (most importantly, including zone) and seemed to produce stable estimates.

In the typical application of the two-part NCI model, usual intake is defined as the probability of consumption on a given day multiplied by the usual amount on consumption days. In this application, we define usual intake as

*U_i_* = (*p_i_*_,_*_low_* × *A_i_*_,_*_low_*) + (*p_i_*_,_*_high_* × *A_i_*_,_*_high_*),

where *A_i_*_,_*_high_* is the usual intake of Vitamin A on high-intake days. For each generated observation in the representative data set, we sampled (with replacement) 100 high-intake recalls from the set of high-intake recalls available from the associated zone, and averaged them as an estimate of *A_i_*_,_*_high_*. This stratified resampling only accounts for the zone covariate, so the resulting distributions (and prevalence estimates) based on the representative sample of the *U_i_* does not fully account for the covariate information. We estimated the standard error for the prevalence of inadequate intake by bootstrapping.

Supplementary Table 1: Estimation of vitamin A intake from breast milk

| Zone | Breast milk intake per day(g/day) | Breast milk vitamin A content (µg retinol/day)(1) | Total vitamin A intake from breast milk (µg RAE/day)(2) |
| --- | --- | --- | --- |
| South | 549 | 1.003 | 551 |
| North | 549 | 0.423 | 232 |
| Yaoundé/ Douala | 549 | 0.862 | 473 |

Supplementary Table 2: Estimation of vitamin B12 intake from breast milk

|  | Breast Milk Intake (g/d)(2) | Breast Milk Intake (ml/d) | Breast Milk B12 concentration (pmol/L) | Breast Milk B12 concentration (pg/ml) | Total B12 intake from breast milk (µg/day) |
| --- | --- | --- | --- | --- | --- |
| South | 549 | 533 | 236 | 319.78 | 0.170 |
| North | 549 | 533 | 47 | 63.69 | 0.034 |
| Yaoundé/ Douala | 549 | 533 | 286 | 387.53 | 0.207 |

*Specific gravity of breast milk is 1.03g/ml: B12 concentration per breast milk: 1 pmol/l = 1.355014 pg/ml*((3)

**Supplementary Table 3: Usual intake distributions assuming different levels of micronutrients added to biscuits, in the presence and absence of large scale fortification program among children 12-59 months of age in Cameroon**

| **Nutrient** | **Fortification level in biscuits**^1^ | **Usual intake per day in the absence of large scale fortification** | | | | | | **Usual intake per day in the presence of f large scale fortification**^2^ | | | | | |
| --- | --- | --- | --- | --- | --- | --- | --- | --- | --- | --- | --- | --- | --- |
|  |  | **South** | | **North** | | **Yaoundé** | | **South** | | **North** | | **Yaoundé** | |
|  |  | **Mean (±SE)** | **Median (P25,p75)** | **Mean**  **(±SE)** | **Median (P25,p75)** | **Mean**  **±SE** | **Median (P25,p75)** | **Mean**  **(±SE)** | **Median**  **(P25,p75)** | **Mean**  **(±SE)** | **Median (P25,p75))** | **Mean**  **(±SE)** | **Median (P25,p75)** |
| **Vitamin A, µg RAE/d** | 0 | 375 ± 25 | 320  (177, 581) | 166 ± 13 | 142  (69, 253) | 247 ± 21 | 142  (69, 253) | 414 ± 29 | 377  (225, 606) | 230 ± 16 | 233  (128, 303) | 399 ± 29 | 375  (208, 590) |
|  | 200 µg RAE | 369 ± 24 | 314  (178, 575) | 169 ± 12 | 147  (72, 256) | 266 ± 21 | 209  (103, 416) | 415 ± 29 | 378  (227, 606) | 234 ± 16 | 233  (131, 307) | 412 ± 29. | 391  (219, 606) |
|  | 350 µg RAE | 369 ± 25 | 315  (180,573) | 172 ± 12 | 151  (75, 258 | 277 ± 21 | 222  (111, 434) | 416 ± 29 | 379  (228, 608) | 236 ± 16 | 233  (132, 310) | 421 ± 30 | 401  (225, 616) |
|  | 500 µg RAE | 371 ± 25 | 317  (182, 574) | 174 ± 13 | 154  (76, 260) | 286 ± 22 | 233  (117, 452) | 418 ± 29 | 381  (229, 610) | 239 ± 16 | 234  (134, 313) | 430 ± 31 | 412  (232, 626) |
|  | 600 µg RAE | 373 ± 25) | 319  (183, 576) | 176 ± 13 | 156  (77, 261) | 293 ± 22 | 239  (120, 465) | 429 ± 29 | 382  (229, 612) | 241 ± 16 | 235  (135, 315) | 436 ± 32 | 417  (234, 634) |
| **Folate, µg DFE/d** | 0 | 137 ± 8 | 128  (89, 177) | 149 ± 9 | 132  (92, 189) | 134 ± 13 | 129  (84, 183) | 305 ± 27 | 267  (165, 410) | 285 ± 28 | 245  (154, 374) | 556 ± 36 | 528  (321, 773) |
|  | 46 µg | 137 ± 8 | 128  (90, 177) | 150 ± 9 | 133  (93, 189) | 140 ± 13 | 135  (88, 192) | 306 ± 27 | 268  (165, 411) | 287 ± 28 | 245  (155, 375) | 561 ± 36 | 532.5  (324, 780) |
|  | 80 µg | 138 ± 8 | 129  (90, 178) | 151 ± 9 | 134  (94, 191) | 144 ± 13 | 139  (90, 197) | 306 ± 27 | 268  (165, 412) | 287 ± 28 | 246  (155, 376) | 565 ± 36 | 536  (326, 784.1) |
|  | 173 µg | 139 ± 8 | 130  (90, 180) | 154 ± 10 | 137  (95, 195) | 153 ± 13 | 148  (96, 210) | 308 ± 28 | 268  (165, 414) | 290 ± 28 | 248  (156, 380) | 575 ± 37 | 545  (330, 798) |
|  | 300 µg | 141 ± 8 | 131  (91, 183) | 157 ± 10 | 140  (96, 200) | 164 ± 13 | 158  (101, 225) | 310 ± 28 | 269  (165, 416) | 293 ± 29 | 250  (157, 384) | 587 ± 38 | 555  (335, 815) |
| **Vitamin B12, absorbable µg/d** | 0 | 1.0 ± 0.2 | 0.7  (0.4, 1.3) | 0.5 ± 0.2 | 0.4  (0.2, 0.7) | 0.9 ± 0.2 | 0.7  (0.3, 1.3) | 1.3 ± 0.2 | 0.8  (0.4, 1.4) | 0.7 ± 0.2 | 0.5  (0.2, 1.0) | 1.8 ± 0.2 | 1.4  (0.7, 2.6) |
|  | 0.74 µg | 1.0 ± 0.2 | 0.7  (0.4, 1.3) | 0.5 ± 0.2 | 0.4  (0.2, 0.7) | 0.9 ± 0.2 | 0.7  (0.3, 1.3) | 1.3 ± 0.2 | 0.9  (0.4, 1.7) | 0.7 ± 0.2 | 0.5  (0.2, 1.0) | 1.8 ± 0.2 | 1.4  (0.7, 2.6) |
|  | 0.98 µg | 1.0 ± 0.2 | 0.7  (0.4, 1.3) | 0.6 ± 0.2 | 0.4  (0.2, 0.7) | 1.0 ± 0.2 | 0.7  (0.3, 1.3) | 1.3 ± 0.2 | 0.9  (0.4, 1.7) | 0.7 ± 0.2 | 0.5  (0.2, 1.0) | 1.8 ± 0.2 | 1.4  (0.7, 2.6) |
|  | 1.2 µg | 1.0 ± 0.2 | 0.7  (0.4, 1.3) | 0.6 ± 0.2 | 0.4(0.2, 0.7) | 1.0 ± 0.2 | 0.7  (0.3, 1.3) | 1.3 ± 0.2 | 0.9  (0.4, 1.7) | 0.7 ± 0.2 | 0.5  (0.2, 1.0) | 1.8 ± 0.2 | 1.4  (0.7, 2.6) |
|  | 2 µg | 1.0 ± 0.2 | 0.7  (0.4, 1.3) | 0.6 ± 0.2 | 0.4(0.2, 0.7) | 1.0 ± 0.2 | 0.7  (0.4, 1.4) | 1.3 ± 0.2 | 0.9  (0.4, 1.7) | 0.8 ± 0.2 | 0.5  (0.2, 1.0) | 1.9 ± 0.2 | 1.5  (0.7, 2.6) |
| **Zinc, mg absorbable zinc/d** | 0 | 0.8 ± 0.0 | 0.8  (0.6, 1.0) | 0.9 ± 0.1 | 1.0  (0.8. 1.2) | 0.7 ± 0.2 | 0.8  (0.6, 1.0) | 0.9 ± 0.0 | 1.0  (0.7, 1.2) | 1.0 ± 0.0 | 1.1  (0.8. 1.3) | 1.1 ± 0.0 | 1.2  (0.9, 1.4) |
|  | 2 mg | 0.8 ± 0.0 | 0.8  (0.6, 1.0) | 0.9 ± 0.2 | 1.0  (0.8. 1.2) | 0.8 ± 0.1 | 0.8  (0.7, 1.0) | 0.9 ± 0.1 | 1.0  (0.7, 1.2) | 1.0 ± 0.0 | 1.1  (0.8. 1.3) | 1.1 ± 0.0 | 1.2  (0.9, 1.4) |
|  | 4 mg | 0.8 ± 0.0 | 0.8  (0.7, 1.0) | 0.9 ± 0.2 | 1.0  (0.8. 1.2) | 0.8 ± 0.1 | 0.8  (0.7, 1.0) | 0.9 ± 0.1 | 1.0  (0.7, 1.2) | 1.0 ± 0.1 | 1.1  (0.8. 1.3) | 1.1 ± 0.0 | 1.2  (0.9, 1.4) |
|  | 5 mg | 0.8 ± 0.0 | 0.8  (0.7, 1.0) | 1.0 ± 0.2 | 1.0  (0.8. 1.2) | 0.8 ± 0.0 | 0.8  (0.7, 1.0) | 0.9 ± 0.0 | 1.0  (0.7, 1.2) | 1.0 ± 0.1 | 1.1  (0.8. 1.3) | 1.1 ± 0.0 | 1.2  (0.9, 1.4) |
|  | 8 mg | 0.8 ± 0.0 | 0.8  (0.7, 1.0) | 1.0 ± 0.2 | 1.0  (0.8. 1.2) | 0.8 ± 0.0 | 0.9  (0.7, 1.0) | 0.9 ± 0.0 | 1.  0(0.7, 1.2) | 1.0 ± 0.1 | 1.1  (0.8. 1.3) | 1.1 ± 0.0 | 1.2  (0.9, 1.4) |
| **Iron, mg absorbable iron/d** | 0 | 0.4 ± 0.0 | 0.3  (0.2, 0.6) | 0.6 ± 0.2 | 0.5  (0.3, 0.8) | 0.4 ± 0.0 | 0.3  (0.1, 0.5) | 0.4 ± 0.0 | 0.3  (0.2, 0.5) | 0.5 ± 0.2 | 0.4  (0.3, 0.6) | 0.3±0.0 | 0.3  (0.1, 0.5) |
|  | 5 mg | 0.4 ± 0.0 | 0.3  (0.2, 0.6) | 0.6 ± 0.0 | 0.5  (0.3, 0.8) | 0.4 ± 0.0 | 0.3  (0.1, 0.5) | 0.4 ± 0.1 | 0.4  (0.2, 0.6) | 0.5 ± 0.0 | 0.5  (0.3, 0.7) | 0.5±0.0 | 0.5  (0.3, 0.7) |
|  | 8 mg | 0.4 ± 0.0 | 0.3  (0.2, 0.6) | 0.6 ± 0.0 | 0.5  (0.3, 0.8) | 0.4 ± 0.0 | 0.3  (0.1,0.5) | 0.4 ± 0.1 | 0.4  (0.2, 0.6) | 0.5 ± 0.0 | 0.5  (0.3, 0.7) | 0.5±0.0 | 0.5  (0.3, 0.7) |
|  | 11 mg | 0.4 ± 0.0 | 0.3  (0.2, 0.6) | 0.6 ± 0.0 | 0.5  (0.3, 0.8) | 0.4 ± 0.0 | 0.3  (0.1, 0.5) | 0.4 ± 0.1 | 0.3  (0.2, 0.6) | 0.5 ± 0.0 | 0.5  (0.3, 0.7) | 0.5±0.0 | 0.5  (0.3, 0.7) |
|  | 15 mg | 0.4 ± 0.0 | 0.3  (0.2, 0.6) | 0.6 ± 0.0 | 0.5  (0.3, 0.8) | 0.4 ± 0.0 | 0.3  (0.1, 0.6) | 0.4 ± 0.1 | 0.4  (0.2, 0.6) | 0.5 ± 0.0 | 0.5  (0.3, 0.7) | 0.5±0.0 | 0.5  (0.3, 0.7) |

Absorbable vitamin B12, absorbable zinc and absorbable iron were estimated using published algorithms, as described in detail in the text(4-6). DFE: Dietary folate Equivalent; RAE: Retinol activity equivalent.

^1^Nutrient fortification level is expressed per 100 gram of biscuits. A fortification level of 0 indicates total nutrient intakes without added micronutrients in biscuits. Values included estimate micronutrient intake from breast milk for children who were breastfed at the time of the survey.

^2^Large scale fortification levels; wheat flour fortification with folic acid (5 mg/kg), vitamin B12 (0.04 mg/kg), zinc (95 mg/kg), iron (60 mg/kg) and edible oil fortification with vitamin A (12 mg/kg).

Supplementary Table 4: Predicting the impact of fortified biscuits, assuming different level of biscuit fortification, on prevalence of inadequate intake among children in the absence and presence of large scale fortification programs

| **Nutrients** | **Fortification level per 100g of biscuits^1^** | **Macro-regions** | | | | | | **National** | |
| --- | --- | --- | --- | --- | --- | --- | --- | --- | --- |
|  |  | **South** | | **North** | | **Yaoundé/Douala** | |  |  |
|  |  | **No LSFF** | **LSFF** | **No LSFF** | **LSFF** | **No LSFF** | **LSFF** | **No LSFF** | **LSFF** |
|  |  | **PI(±SE)** | **PI(±SE)** | **PI(±SE)** | **PI(SE±)** | **PI(±SE)** | **PI(±SE)** | **PI(±SE)** | **PI(±SE)** |
| **Vitamin A** | 0 | 29.2 ± 8.4 | 19.8 ± 9.3 | 62.3 ± 4.2 | 47.3 ± 4.9 | 51.4 ± 5.4 | 21.5 ± 8.9 | 45.1 ± 4.4 | 30.9 ± 6.1 |
|  | 200 µg RAE | 29.2 ± 8.9 | 19.5 ± 9.8 | 61.8 ± 4.2 | 46.6 ± 4.9 | 46.9 ± 6.3 | 19.8 ± 8.6 | 44.3 ± 4.4 | 30.2 ± 6.1 |
|  | 350 µg RAE | 29.0 ± 9.1 | 19.4 ± 9.8 | 61.3 ± 4.0 | 46.0 ± 4.9 | 44.4 ± 6.8 | 19.0 ± 8.4 | 44.3 ± 4.4 | 29.7 ± 6.3 |
|  | 500 µg RAE | 28.5 ± 9.3 | 19.2 ± 9.8 | 60.8 ± 4.0 | 45.6 ± 4.9 | 42.4 ± 7.5 | 18.2 ± 8.4 | 43.6 ± 4.4 | 29.4 ± 6.3 |
|  | 600 µg RAE | 28.3 ± 9.3 | 19.2 ± 9.8 | 60.4 ± 4.0 | 45.3 ± 4.9 | 41.4 ± 7.5 | 17.9 ± 8.2 | 43.1 ± 4.4. | 29.2 ± 6.3 |
| **Folate** | 0 | 41.2 ± 5.8 | 8.5 ± 6.8 | 45.1 ± 4.7 | 15.5 ± 7.2 | 42.3 ± 8.2 | 9.0 ± 2.3 | 42.9 ± 4.2 | 11.4 ± 5.6 |
|  | 46µg | 41.0 ± 5.6 | 8.5 ± 6.8 | 44.0 ± 4.9 | 15.5 ± 7.2 | 38.7 ± 7.7 | 9.0 ± 2.3 | 41.8 ± 4.2 | 11.4 ± 5.6 |
|  | 80µg | 40.8 ± 5.6 | 8.5 ± 6.8 | 43.6 ± 4.9 | 15.5 ± 7.2 | 37.0 ± 7.7 | 9.0 ± 2.3 | 41.3 ± 4.2 | 11.4 ± 5.6 |
|  | 173µg | 40.2 ± 5.4 | 8.5 ± 6.8 | 42.3 ± 4.9 | 15.5 ± 7.0 | 32.9 ± 7.0 | 9.0 ± 2.3 | 39.8 ± 4.2 | 11.4 ± 5.4 |
|  | 300µg | 39.3 ± 5.4 | 8.5 ± 6.8 | 40.8 ± 5.1 | 15.4 ± 7.0 | 29.1 ± 6.3 | 9.0 ± 2.3 | 38.1 ± 4.0 | 11.4 ± 5.4 |
| **Vitamin B12** | 0 | 18.4 ± 11.2 | 14.6 ± 7.7 | 48.7 ± 10.3 | 39.2 ± 8.8 | 22.1 ± 10.0 | 11.6 ± 3.7 | 30.9 ± 9.9 | 23.8 ± 6.5 |
|  | 0.74µg | 18.4 ± 11.0 | 14.6 ± 7.6 | 48.7 ± 10.0 | 39.0 ± 8.8 | 21.4 ± 9.6 | 11.5 ± 3.6 | 30.9 ± 9.6 | 23.6 ± 6.6 |
|  | 0.98µg | 18.4 ± 11.0 | 14.6 ± 7.5 | 48.5 ± 10.0 | 38.9 ± 8.6 | 21.3 ± 9.3 | 11.4 ± 3.6 | 30.9 ± 9.5 | 23.6 ± 6.4 |
|  | 1.2µg | 18.4 ± 11.0 | 14.6 ± 7.6 | 48.4 ± 10.0 | 38.8 ± 8.8 | 21.1 ± 9.1 | 11.4 ± 3.6 | 30.9 ± 9.4 | 23.5 ± 6.5 |
|  | 2µg | 18.4 ± 10.7 | 14.5 ± 7.5 | 48.0 ± 4.9 | 38.6 ± 8.7 | 20.5 ± 8.9 | 11.3 ± 3.5 | 30.7 ± 9.3 | 23.4 ± 6.5 |
| **Zinc** | 0 | 42.4 ± 4.4 | 21.3 ± 4.2 | 27.2 ± 5.6 | 20.4 ± 4.0 | 44.3 ± 6.8 | 11.2 ± 3.0 | 36.8 ± 2.8 | 19.2 ± 1.2 |
|  | 2mg | 41.9 ± 3.7 | 21.1 ± 4.2 | 27.1 ± 5.1 | 20.2 ± 4.0 | 39.9 ± 6.1 | 11.0 ± 3.0 | 35.8 ± 2.6 | 19.0 ± 3.0 |
|  | 4mg | 41.4 ± 3.5 | 21.1 ± 4.2 | 26.8 ± 4.9 | 20.0 ± 4.0 | 36.8 ± 6.3 | 10.9 ± 3.0 | 34.9 ± 2.8 | 18.9 ± 3.0 |
|  | 5mg | 41.2 ± 3.5 | 21.0 ± 4.2 | 26.6 ± 4.9 | 19.9 ± 4.0 | 35.6 ± 6.1 | 10.9 ± 2.8 | 34.5 ± 2.8 | 18.8 ± 2.8 |
|  | 8mg | 40.6 ± 3.5 | 21.0 ± 4.2 | 26.1 ± 4.7 | 19.7 ± 4.0 | 32.6 ± 6.3 | 10.7 ± 2.8 | 33.5 ± 2.8 | 18.7 ± 3.0 |
| **Iron** | 0 | 74.6 ± 1.9 | 71.7 ± 1.9 | 59.4 ± 1.2 | 56.8 ± 3.0 | 76.7 ± 1.2 | 68.8 ± 2.6 | 69.9 ± 1.4 | 66.1 ± 1.4 |
|  | 5mg/ | 74.5 ± 1.9 | 71.7 ± 1.9 | 58.9 ± 3.0 | 56.6 ± 3.0 | 75.5 ± 2.6 | 68.4 ± 2.6 | 69.5 ± 1.4 | 66.0 ± 1.4 |
|  | 8mg | 74.4 ± 1.9 | 71.6 ± 1.9 | 58.7 ± 3.0 | 56.5 ± 3.0 | 75.2 ± 2.6 | 68.3 ± 2.6 | 69.3 ± 1.4 | 65.8 ± 1.4 |
|  | 11mg | 74.4 ± 1.9 | 71.6 ± 1.9 | 58.5 ± 3.0 | 56.4 ± 3.0 | 74.9 ± 2.6 | 68.2 ± 2.6 | 69.1 ± 1.4 | 65.8 ± 1.4 |
|  | 15mg | 74.3 ± 1.9 | 71.6 ± 1.9 | 58.3 ± 3.0 | 56.3 ± 3.0 | 74.9 ± 2.6 | 68.2 ± 2.6 | 69.0 ± 1.4 | 65.7 ± 1.4 |

PI: Prevalence of inadequacy: % usual intake below the Physiological or Estimated Average Requirements, SE: standard error (%).

Prevalence of inadequate intake for vitamin A and folate was estimated based on estimated average requirement. Physiological requirements values were applied to estimate the prevalence of inadequate for vitamin B12, zinc and iron from the absorbable nutrient intake. Zinc estimates are based on Institute of Medicine (IOM) physiological requirements(7). Absorbable vitamin B12, absorbable zinc and absorbable iron were estimated using published algorithms, as described in detail in the text(4-6).

^1^Nutrient fortification level is expressed per 100 gram of biscuits. A fortification level of 0 indicates total nutrient intakes without added micronutrients in biscuits. RAE: Retinol activity equivalent.

^“^No LSFF” refers to prevalence from natural food sources, in the absence of a large-scale food fortification (LSFF) program. LSFF levels; wheat flour fortification with folic acid (5 mg/kg), vitamin B12 (0.04 mg/kg), zinc (95 mg/kg), iron (60 mg/kg) and edible oil fortification with vitamin A (12 mg/kg).

Effective coverage was calculated by subtracting the prevalence of inadequate intake at each fortification level from the baseline (without added micronutrients in biscuits)

**Supplementary Table 5: Sensitivity analysis to compare the prevalence of inadequacy of vitamin B12 for preschool children estimated based on total dietary vitamin B12 intake and absorbable vitamin B12 at different level of biscuits fortification**

| **Vitamin B12**  **Fortification**  **Level**^1^ | **Macro-region** | | | | | | **National** | | |
| --- | --- | --- | --- | --- | --- | --- | --- | --- | --- |
|  | **South** | | **North** | | **Yaoundé/Douala** | |  |  |  |
|  | **Total dietary intake (%)**  **± SE** | **Absorbable**  **(%)**  **± SE** | **Total dietary intake (%)±SE** | **Absorbable (%)± SE** | **Total dietary intake (%)**  **± SE** | **Absorbable (%)±SE** | **Total dietary intake (%)**  **± SE** | **Absorbable (%)**  **± SE** | |
| 0 | 13.3 ± 9.3 | 18.4 ± 11.2 | 38.9 ± 11.7 | 48.7 ± 10.3 | 17.5 ± 8.4 | 22.1 ± 10.0 | 24.2 ± 9.6 | 30.9 ± 9.9 |  |
| 0.74 µg | 13.3 ± 9.3 | 18.4 ± 11.0 | 38.9 ± 11.4 | 48.7 ± 10.0 | 17.1 ± 7.9 | 21.4 ± 9.6 | 24.2 ± 9.3 | 30.9 ± 9.6 |  |
| 0.98 µg | 13.3 ± 9.3 | 18.4 ± 11.0 | 38.8 ± 11.4 | 48.5 ± 10.0 | 17.0 ± 7.7 | 21.3 ± 9.3 | 24.2 ± 9.3 | 30.9 ± 9.5 |  |
| 1.2 µg | 13.3 ± 9.3 | 18.4 ± 11.0 | 38.7 ± 11.4 | 48.4 ± 10.0 | 16.9 ± 7.7 | 21.1 ± 9.1 | 24.1 ± 9.3 | 30.9 ± 9.4 |  |
| 2 µg | 13.3 ± 9.1 | 18.4 ± 10.7 | 38.3 ± 11.2 | 48.0 ± 4.9 | 16.5 ± 7.5 | 20.5 ± 8.9 | 23.9 ± 9.1 | 30.7 ± 9.3 |  |

Total dietary intake (%): Prevalence of vitamin B12 intake below Estimated Average Requirements.

Absorbable (%): Prevalence of vitamin B12 usual intake below physiological requirements, SE: standard error (%).

Absorbable vitamin B12 intake were estimated using published algorithm, as described in detail in the text(6)

^1^Vitamin B12 fortification level is expressed per 100 gram of biscuits. A fortification level of 0 indicates total nutrient intakes without added vitamin B12 in biscuits.

Effective coverage was calculated by subtracting the prevalence of inadequate intake at each fortification level from the baseline (without added micronutrients in biscuits)

**Supplementary Table 6: Sensitivity analysis to compare the prevalence of inadequate zinc intake based on cutoff values recommended by different expert groups**

| **Zinc cutoffs** | **Fortification level per 100g^1^** | **Macro-regions** | | | | | | | **National** | |
| --- | --- | --- | --- | --- | --- | --- | --- | --- | --- | --- |
|  |  | **South** | | **North** | | **Yaoundé/Douala** | | |  |  |
|  |  | **No LSFF** | **LSFF** | **No LSFF** | **LSFF** | **No LSFF** | | **LSFF** | **No LSFF** | **LSFF** |
|  |  | **PI(±SE)** | **PI(±SE)** | **PI(±SE)** | **PI(SE±)** | **PI(±SE)** | | **PI(±SE)** | **PI(±SE)** | **PI(±SE)** |
| **IZiNCG**^2^ | 0 | 5.8 ± 6.8 | 5.1 ± 2.8 | 5.8 ± 3.0 | 5.5 ± 3.0 | 11.5 ± 4.4 | | 9.0 ± 2.6 | 6.6 ± 4.7 | 6.2 ± 1.9 |
|  | 2mg | 5.8 ± 6.2 | 5.0 ± 2.6 | 5.8 ± 2.8 | 5.5 ± 2.8 | 11.3 ± 3.7 | | 9.0 ± 2.33 | 6.6 ± 4.2 | 6.2 ± 1.9 |
|  | 4mg | 5.8 ± 5.9 | 5.0 ± 2.6 | 5.8 ± 2.8 | 5.5 ± 2.8 | 11.0 ± 3.5 | | 9.0 ± 2.33 | 6.6 ± 3.7 | 6.2 ± 1.9 |
|  | 5mg | 5.8 ± 5.6 | 5.0 ± 2.6 | 5.8 ± 2.8 | 5.5 ± 2.8 | 11.0 ± 3.3 | | 9.0 ± 2.33 | 6.6 ± 3.7 | 6.1 ± 1.9 |
|  | 8mg | 5.8 ± 5.1 | 5.0 ± 2.6 | 5.8 ± 2.6 | 5.5 ± 2.6 | 10.7 ± 3.0 | | 9.0 ± 2.33 | 6.6 ± 3.3 | 6.1 ± 1.9 |
| **EFSA**^3^ | 0 | 87.2 ± 7.9 | 57.1 ± 4.9 | 65.9 ± 4.9 | 51.9 ± 4.4 | 84.8 ± 6.1 | 34.5 ± 5.1 | | 78.4 ± 3.7 | 51.2 ± 2.8 |
|  | 2mg | 86.0 ± 7.7 | 57.0 ± 4.9 | 65.5 ± 4.4 | 51.6 ± 4.4 | 82.0 ± 5.1 | 33.7 ± 4.9 | | 77.3 ± 4.0 | 50.9 ± 2.8 |
|  | 4mg | 85.4 ± 7.5 | 56.9 ± 4.9 | 65.2 ± 4.2 | 51.4 ± 4.4 | 79.9 ± 4.7 | 33.0 ± 4.9 | | 76.5 ± 3.7 | 50.6 ± 2.8 |
|  | 5mg | 85.1 ± 7.2 | 56.9 ± 4.9 | 65.0 ± 4.0 | 51.3 ± 4.4 | 79.1 ± 4.7 | 32.8 ± 4.9 | | 76.2 ± 3.7 | 50.5 ± 2.8 |
|  | 8mg | 84.5 ± 7.0 | 56.7 ± 4.9 | 64.5 ± 4.0 | 51.0 ± 4.7 | 76.8 ± 4.7 | 32.1 ± 4.9 | | 75.3 ± 3.7 | 50.2 ± 2.8 |
| **IZiNCG EAR**^4^ | 0 | 10.4 ± 6.8 | 3.6 ± 4.9 | 9.3 ± 3.0 | 6.6 ± 4.4 | 12.6 ± 4.0 | 8.8 ± 2.6 | | 10.4 ± 3.5 | 5.8 ± 3.7 |
|  | 2mg | 10.3 ± 8.4 | 3.6 ± 4.9 | 8.8 ± 2.8 | 6.6 ± 4.4 | 11.2 ± 3.7 | 8.8 ± 2.6 | | 9.9 ± 40 | 5.8 ± 3.7 |
|  | 4mg | 9.2 ± 8.9 | 3.6 ± 4.9 | 8.6 ± 3.3 | 6.6 ± 4.4 | 10.7 ± 3.5 | 8.8 ± 2.6 | | 9.2 ± 4.4 | 5.8 ± 3.7 |
|  | 5mg | 7.6 ± 8.9 | 3.6 ± 4.9 | 8.2 ± 3.0 | 6.6 ± 4.4 | 9.7 ± 3.7 | 8.8 ± 2.6 | | 8.2 ± 4.4 | 5.8 ± 3.7 |
|  | 8mg | 4.9 ±10.7 | 3.6 ± 4.9 | 6.7 ± 3.0 | 6.6 ± 4.4 | 9.7 ± 2.6 | 8.8 ± 2.5 | | 6.4 ± 5.4 | 5.8 ± 3.7 |
| **EFSA EAR**^5^ | 0 | 45.7 ± 9.3 | 18.2 ± 8.9 | 37.0 ± 6.3 | 20.4 ± 7.9 | 41.1 ± 8.6 | 9.9 ± 8.2 | | 41.5 ± 4.4 | 17.7 ± 6.7 |
|  | 2mg | 45.0 ± 8.2 | 18.2 ± 8.9 | 35.9 ± 6.1 | 20.4 ± 7.7 | 39.7 ± 8.6 | 9.8 ± 8.2 | | 40.5 ± 5.1 | 17.6 ± 6.7 |
|  | 4mg | 44.5 ± 8.2 | 18.2 ± 9.1 | 34.8 ± 6.8 | 20.3 ± 7.7 | 35.8 ± 7.9 | 9.8 ± 7.5 | | 39.2 ± 5.4 | 17.6 ± 6.7 |
|  | 5mg | 44.2 ± 8.2 | 18.2 ± 9.1 | 34.2 ± 6.8 | 20.2 ± 7.5 | 34.7 ± 8.9 | 9.7 ± 7.5 | | 38.7 ± 5.8 | 17.6 ± 6.8 |
|  | 8mg | 43.0 ± 7.7 | 18.2 ± 6.7 | 31.3 ± 5.8 | 20.0 ± 7.5 | 29.2 ± 9.3 | 9.6 ± 6.7 | | 36.0 ± 4.7 | 17.5 ± 6.8 |

PI: Prevalence of inadequacy: % usual intake below the physiological or Estimated Average Requirements, SE: standard error (%).

^“^No LSFF” refers to prevalence inadequacy from natural food sources, in the absence of a large-scale food fortification (LSFF) program. Large scale fortification levels; wheat flour fortification with zinc: 95 mg/kg.

Effective coverage was calculated by subtracting the prevalence of inadequate intake at each fortification level from the baseline (without added micronutrients in biscuits).

^1^Zinc fortification level is expressed per 100 gram of biscuits. A fortification level of 0 indicates total nutrient intakes without added zinc in biscuits.

^2^ IZiNCG physiological requirement cut-offs were applied on absorbable zinc to estimate prevalence of inadequate intake

^3^ EFSA physiological requirement cut-offs were applied on absorbable zinc to estimate prevalence of inadequate intake

^4^ IZiNCG Estimated Average Requirement were applied on total zinc intake, assuming the diet is high in phytate, to estimate prevalence of inadequate intake

^5^ EFSA Estimated Average Requirement were applied on total zinc intake, assuming the diet is high in phytate, to estimate prevalence of inadequate intake (7)

**Supplementary Table 7: Usual nutrient intake distribution assuming different level of biscuit fortification in the presence and absence of large scale fortification program among women in reproductive age in Cameroon**

| **Nutrients** | **Fortification level in biscuits**^1^ | **Usual intake per day in the absence of large scale fortification** | | | | | | **Usual intake per day in the presence of f large scale fortification**^2^ | | | | | |
| --- | --- | --- | --- | --- | --- | --- | --- | --- | --- | --- | --- | --- | --- |
|  |  | **South** | | **North** | | **Yaoundé** | | **South** | | **North** | | **Yaoundé** | |
|  |  | **Mean (±SE)** | **Median (P25,p75)** | **Mean**  **(±SE)** | **Median (P25,p75)** | **Mean**  **(±SE)** | **Median (P25,p75)** | **Mean**  **(±SE)** | **Median**  **(P25,p75)** | **Mean**  **(±SE)** | **Median**  **(P25,p75)** | **Mean**  **(±SE)** | **Median**  **(P25,p75)** |
| **Vitamin A, µg RAE/d** | 0 | 741 ± 65. | 769  (642, 877) | 239 ± 27 | 230  (169, 304) | 499 ± 52 | 531  (436, 622) | 775 ± 66 | 772  (602, 952) | 382 ± 38 | 369  (272, 481) | 832 ± 57 | 871  (676, 1064) |
|  | 200 µg RAE | 742 ± 65 | 781  (668, 878) | 245 ± 27 | 240  (175, 311) | 509 ± 53 | 555  (462, 626) | 777 ± 66 | 773  (602, 955) | 387 ± 38 | 373  (274, 488) | 838 ± 56 | 876  (678, 1073) |
|  | 350 µg RAE | 743 ± 65 | 784  (672, 879) | 250 ± 27 | 245  (176, 317) | 516 ± 54 | 566  (469, 634) | 778 ± 66 | 774  (603, 956) | 391 ± 38 | 376  (276, 493) | 845 ± 58 | 882  (682, 1082) |
|  | 500 µg RAE | 745 ± 65 | 786  (673, 882) | 252 ± 27 | 248  (177, 321) | 522 ± 55 | 573  (473, 643) | 780 ± 66 | 775  (602, 960) | 394 ± 38 | 379  (277, 497) | 850 ± 58 | 887  (684, 1090) |
|  | 600 µg RAE | 747 ± 65 | 789  (675, 883) | 255 ± 27 | 250  (178, 323) | 525 ± 55 | 578  (475, 647) | 784 ± 66 | 777  (604, 961) | 396 ± 38 | 381  (278, 500) | 855 ± 60 | 892(689, 1095) |
| **Folate, µg DFE/d** | 0 | 276 ± 14 | 279  (219, 344) | 356 ± 23 | 351  (279, 430) | 240 ± 16 | 255  (200, 310) | 475 ± 25 | 441  (292, 627) | 604 ± 49 | 556  (385, 771) | 782 ± 51 | 761  (500, 1062) |
|  | 46ug | 277 ± 14 | 281  (220, 344) | 358 ± 23 | 353  (282, 432) | 243 ± 16 | 259  (204, 313) | 476 ± 25 | 441  (293, 628) | 606 ± 49 | 558  (387, 773) | 784 ± 51 | 763  (502, 1065) |
|  | 80ug | 277 ± 14 | 282  (221, 345) | 360 ± 24 | 354.9  (283, 433) | 245 ± 16 | 262  (207, 315) | 477 ± 25 | 442  (293, 628) | 608 ± 48 | 559  (388, 775) | 786 ± 52 | 766  (504, 1068) |
|  | 173ug | 279 ± 14 | 284  (224, 346) | 364 ± 24 | 359  (288, 438) | 249 ± 16 | 268  (212, 320) | 478 ± 25 | 443  (294, 630) | 612 ± 49 | 563  (390,780) | 791 ± 53 | 770(506, 1075) |
|  | 300ug | 281 ± 14 | 288  (227, 348) | 369 ± 25 | 365  (294, 443) | 254 ± 17.0 | 276  (219 ,327) | 480 ± 25 | 445  (296, 632) | 617 ± 49. | 567  (393,786) | 799 ± 54 | 778  (512, 1086) |
| **Vitamin B12, absorbable µg/d** | 0 | 5.0 ± 0.5 | 3.9  (2.0, 6.7) | 3.3 ± 0.5 | 2.4  (2.2, 4.4) | 5.6 ± 0.5 | 5.0  (2.7, 8.0) | 6.0 ± 0.5 | 4.4  (2.1, 8.1) | 4.2 ± 0.5 | 3.0  (1.4,5.7) | 9.0 ± 0.9 | 7.4  (3.5, 12.8) |
|  | 0.74ug | 5.0 ± 0.5 | 4.0  (2.2, 6.7) | 3.4 ± 0.5 | 2.6  (1.4, 4.4) | 5.8 ± 0.5 | 4.9  (2.5, 8.1) | 6.0 ± 0.5 | 4.4  (2.1, 8.1) | 4.3 ± 0.5 | 3.0  (1.4,5.7) | 9.1 ± 0.7 | 7.4  (3.5, 12.9) |
|  | 0.98ug | 5.0 ± 0.5 | 4.0  (2.2, 6.7) | 3.4 ± 0.5 | 2.6  (1.4, 4.5) | 5.8 ± 0.5 | 4.9  (2.5, 8.1) | 6.0 ± 0.5 | 4.4  (2.1, 8.1) | 4.3 ± 0.5 | 3.0  (1.4, 5.7) | 9.1 ± 0.7 | 7.4  (3.5, 12.9) |
|  | 1.2ug | 5.0 ± 0.5 | 4.0  (2.2, 6.7) | 3.4 ± 0.5 | 2.6  (1.4, 4.5) | 5.8 ± 0.5 | 4.9  (2.5, 8.1) | 6.0 ± 0.5 | 4.4  (2.1, 8.1) | 4.3 ± 0.5 | 3.0  (1.4, 5.7) | 9.1±0.7 | 7.4  (3.5, 12.9) |
|  | 2ug | 5.0 ± 0.5 | 4.0  (2.2, 6.7) | 3.4 ± 0.5 | 2.6  (1.4, 4.5) | 5.9 ± 0.5 | 4.9  (2.5, 8.2) | 6.0 ± 0.5 | 4.4  (2.1, 8.2) | 4.3 ± 0.5 | 3.0  (1.4, 5.7) | 9.1±0.7 | 7.4  (3.5, 12.9) |
| **Zinc, mg absorbable zinc/d** | 0 | 1.2 ± 0.2 | 1.2  (0.9, 1.4) | 2.2 ± 0.2 | 2.2  (1.8, 2.7) | 1.1 ± 0.2 | 1.2  (0.9, 1.5) | 1.6 ± 0.2 | 1.5  (1.1, 2.0) | 2.8 ± 0.2 | 2.7  (2.1, 3.4) | 2.2 ± 0.2 | 2.3  (1.6, 2.9) |
|  | 2mg | 1.2 ± 0.2 | 1.2  (0.9, 1.4) | 2.2 ± 0.2 | 2.  2(1.8, 2.7) | 1.2 ± 0.2 | 1.2  (0.9, 1.5) | 1.6 ± 0.2 | 1.5  (1.1, 2.0) | 2.8 ± 0.2 | 2.7  (2.1, 3.4) | 2.2 ± 0.2 | 2.3  (1.6, 3.0) |
|  | 4mg | 1.2 ± 0.2 | 1.2  (0.9, 1.4) | 2.3 ± 0.2 | 2.2  (1.8, 2.7) | 1.2 ± 0.2 | 1.2  (0.9, 1.5) | 1.6 ± 0.2 | 1.5  (1.1, 2.0) | 2.8 ± 0.2 | 2.7  (2.1, 3.4) | 2.2 ± 0.2 | 2.3  (1.6, 3.0) |
|  | 5mg | 1.2 ± 0.2 | 1.2  (0.9, 1.4) | 2.3 ± 0.2 | 2.2  (1.8, 2.7) | 1.2 ± 0.2 | 1.2  (0.9, 1.5) | 1.6 ± 0.2 | 1.5  (1.1, 2.0) | 2.8 ± 0.2 | 2.7  (2.1, 3.5) | 2.2 ± 0.2 | 2.3  (1.7, 3.0) |
|  | 8mg | 1.2 ± 0.2 | 1.2  (0.9, 1.4) | 2.3 ± 0.2 | 2.2  (1.8, 2.7) | 1.2 ± 0.2 | 1.2  (0.9, 1.5) | 1.6 ± 0.2 | 1.5  (1.1, 2.0) | 2.8 ± 0.2 | 2.7  (2.1, 3.5) | 2.3 ± 0.2 | 2.3  (1.7, 3.0) |
| **Iron, mg absorbable iron/d** | 0 | 0.5 ± 0.2 | 0.5  (0.3, 0.7) | 0.7 ± 0.2 | 0.7  (0.5, 1.0) | 0.5 ± 0.0 | 0.5  (0.3, 0.8) | 0.5 ± 0.0 | 0.5  (0.3, 0.7) | 0.7 ± 0.0 | 0.7  (0.5, 0.9) | 0.4 ± 0.2 | 0.4  (0.2, 0.6) |
|  | 5mg | 0.5 ± 0.2 | 0.5  (0.3, 0.7) | 0.7 ± 0.2 | 0.7  (0.5, 1.0) | 0.5 ± 0.0 | 0.5  (0.3, 0.8) | 0.6 ± 0.0 | 0.5  (0.3, 0.7) | 0.7 ± 0.2 | 0.7  (0.5, 1.0) | 0.6 ± 0.0 | 0.6  (0.3, 0.8) |
|  | 8mg | 0.5 ± 0.2 | 0.5  (0.3, 0.7) | 0.7 ± 0.2 | 0.7  (0.5, 1.0) | 0.5 ± 0.0 | 0.5  (0.3, 0.8) | 0.6 ± 0.0 | 0.5  (0.3, 0.7) | 0.7 ± 0.2 | 0.7  (0.5, 1.0) | 0.6 ± 0.0 | 0.6  (0.3, 0.8) |
|  | 11mg | 0.5 ± 0.2 | 0.5  (0.3, 0.7) | 0.7 ± 0.2 | 0.7  (0.5, 1.0) | 0.5 ± 0.0 | 0.5  (0.3, 0.8) | 0.6 ± 0.0 | 0.5  (0.3, 0.7) | 0.7 ± 0.2 | 0.7  (0.5, 1.0) | 0.6 ± 0.0 | 0.6  (0.3, 0.8) |
|  | 15mg | 0.5 ± 0.2 | 0.5  (0.3, 0.7) | 0.7 ± 0.2 | 0.7  (0.5, 1.0) | 0.5 ± 0.0 | 0.5  (0.3, 0.8) | 0.6 ± 0.0 | 0.5  (0.3, 0.7) | 0.7 ± 0.0 | 0.7  (0.5, 1.0) | 0.6 ± 0.0 | 0.6  (0.3, 0.8) |

Absorbable vitamin B12, absorbable zinc and absorbable iron were estimated using published algorithms, as described in detail in the text (5, 6, 8). DFE, dietary folate equivalent; RAE, retinol activity equivalent.

^1^Nutrient fortification level is expressed per 100 gram of biscuits. A fortification level of 0 indicates total nutrient intakes without added micronutrients in biscuits. ^2^Large scale fortification levels; wheat flour fortification with folic acid (5 mg/kg), vitamin B12 (0.04 mg/kg), zinc (95 mg/kg), iron (60 mg/kg) and edible oil fortification with vitamin A (12 mg/kg). **Supplementary Table 8: Predicting the impact of fortified biscuits, assuming different level of biscuit fortification, on prevalence of inadequate intake among women in reproductive age in the absence and presence of large scale fortification programs**

| **Nutrients** | **Fortification level per 100g biscuits^1^** | **Macro-regions** | | | | | | **National** | |
| --- | --- | --- | --- | --- | --- | --- | --- | --- | --- |
|  |  | **South** | | **North** | | **Yaoundé/Douala** | |  |  |
|  |  | **No LSFF** | **LSFF** | **No LSFF** | **LSFF** | **No LSFF** | **LSFF** | **No LSFF** | **LSFF** |
|  |  | **PI(±SE)** | **PI(±SE)** | **PI(±SE)** | **PI(SE±)** | **PI(±SE)** | **PI(±SE)** | **PI(±SE)** | **PI(±SE)** |
| **Vitamin A** | 0 | 61.4 ± 10.8 | 36.2 ± 15.5 | 80.5 ± 9.7 | 59.1 ± 14.7 | 60.0 ± 10.5 | 29.0 ± 13.7 | 64.5 ± 10.3 | 39.6 ± 14.6 |
|  | 200 µg RAE | 60.4 ±10.8 | 35.6 ± 17.6 | 80.0 ± 9.9 | 58.9 ± 15.6 | 59.9 ± 10.5 | 28.9 ± 16.2 | 64.5 ± 10.4 | 38.6 + 16.5 |
|  | 350 µg RAE | 60.0 ± 11.0 | 35.3 ± 15.1 | 79.5 ± 10.0 | 57.3 ± 14.7 | 59. 6 ± 10.5 | 28.5 ± 13.4 | 64.5 ± 10.5 | 38.6 ± 14.4 |
|  | 500 µg RAE | 59.8 ± 9.8 | 35.2 ± 15.4 | 77.3 ± 9.5 | 56.0 ± 14.9 | 59.2 ± 9.1 | 28.4 ± 13.7 | 64.5 ± 9.5 | 38.4 ± 14.7 |
|  | 600 µg RAE | 59.8 ± 10.3 | 35.2 ± 15.2 | 77.3 ± 9.6 | 55.1 ± 14.8 | 59.2 ± 9.5 | 28.4 ± 13.7 | 63.2 ± 9.8 | 38.1 ± 14.6 |
| **Folate** | 0 | 80.9 ± 6.5 | 38.9 ± 4.7 | 52.7 ± 8.6 | 24.5 ± 6.8 | 85.7 ± 11.4 | 17.5 ± 3.7 | 72.6 ± 5.1 | 29.7 ± 4.2 |
|  | 46µg | 80.9 ± 6.5 | 38.8 ± 4.7 | 51.9 ± 8.6 | 24.3 ± 6.8 | 84.8 ± 11.7 | 17.4 ± 3.7 | 72.2 ± 5.4 | 29.5 ± 4.2 |
|  | 80µg | 80.9 ± 6.3 | 38.7 ± 4.9 | 51.4 ± 8.6 | 24.2 ± 6.8 | 84.3 ± 11.2 | 17.4 ± 3.7 | 71.9 ± 5.1 | 29.4 ± 4.2 |
|  | 173µg | 80.5 ± 6.7 | 38.5 ± 4.9 | 50.0 ± 8.9 | 23.9 ± 7.0 | 82.8 ± 12.1 | 17.3 ± 3.7 | 71.0 ± 5.6 | 29.2 ± 4.2 |
|  | 300µg | 80.0 ± 7.7 | 38.2 ± 4.9 | 48.2 ± 9.32 | 23.6 ± 7.0 | 81.1 ± 12.8 | 17.2 ± 3.7 | 69.8 ± 5.8 | 28.9 ± 4.4 |
| **Vitamin B12** | 0 | 22.1 ± 12.4 | 22.1 ± 7.0 | 38.7 ± 13.3 | 37.2 ± 8.2 | 19.9 ± 8.4 | 17.6 ± 4.2 | 27.1 ± 11.2 | 27.1 ± 5.8 |
|  | 0.74µg | 22.1 ± 12.4 | 22.1 ± 7.0 | 38.7 ± 12.4 | 37.0 ± 8.2 | 19.9 ± 8.4 | 17.5 ± 4.2 | 27.1 ± 11.2 | 27.1 ± 5.8 |
|  | 0.98µg | 22.1 ± 11.9 | 22.1 ± 7.0 | 38.7 ± 12.4 | 37.0 ± 8.2 | 19.9 ± 8.4 | 17.5 ± 4.2 | 27.1 ± 10.7 | 27.1 ± 5.8 |
|  | 1.2µg | 22.1 ± 11.9 | 22.1 ± 7.0 | 38.7 ± 12.1 | 37.0 ± 8.2 | 19.9 ± 8.4 | 17.5 ± 4.2 | 27.1 ± 10.7 | 27.1 ± 5.8 |
|  | 2µg | 22.1 ± 11.7 | 22.1 ± 7.0 | 38.7 ± 11.9 | 36.7 ± 8.2 | 19.9 ± 8.4 | 17.5 ± 4.2 | 27.1 ± 10.5 | 27.1 ± 5.8 |
| **Zinc** | 0 | 100 ± 0.2 | 97.2 ± 1.4 | 88.7 ± 4.2 | 67.1 ± 4.2 | 99.9 ± 0.2 | 78.8 ± 4.2 | 96.3 ± 1.4 | 83.4 ± 2.1 |
|  | 2mg | 100 ± 0.2 | 97.2 ± 1.6 | 88.5 ± 4.4 | 66.7 ± 4.2 | 99.9 ± 0.2 | 78.4 ± 4.2 | 96.2 ± 1.4 | 83.2 ± 2.1 |
|  | 4mg | 100 ± 0.2 | 97.1 ± 1.6 | 88.2 ± 4.4 | 66.3 ± 4.2 | 99.9 ± 0.2 | 78.0 ± 4.4 | 96.1 ± 1.4 | 83.0 ± 2.1 |
|  | 5mg | 100 ± 0.2 | 97.1 ± 1.6 | 88.1 ± 4.4 | 66.2 ± 4.2 | 99.9 ± 0.2 | 77.8 ± 4.4 | 96.1 ± 1.6 | 82.9 ± 2.3 |
|  | 8mg | 100 ± 0.2 | 97.1 ± 1.6 | 87.6 ± 4.7 | 65.7 ± 4.2 | 99.9 ± 0.2 | 77.3 ± 4.4 | 95.9 ± 1.6 | 82.6 ± 2.3 |
| **Iron** | 0 | 96.9 ± 0.5 | 96.9 ± 0.5 | 91.1 ± 1.6 | 91.1 ± 1.2 | 96.4 ± 0.5 | 96.4 ± 0.2 | 94.9 ± 0.7 | 94.9 ± 0.5 |
|  | 5mg/ | 96.9 ± 0.5 | 96.9 ± 0.5 | 91.1 ± 1.6 | 91.1 ± 1.6 | 96.4 ± 0.5 | 96.4 ± 0.7 | 94.9 ± 0.7 | 94.9 ± 0.7 |
|  | 8mg | 96.9 ± 0.5 | 96.9 ± 0.5 | 91.1 ± 1.6 | 91.1 ± 1.6 | 96.4 ± 0.5 | 96.4 ± 0.7 | 94.9 ± 0.7 | 94.9 ± 0.7 |
|  | 11mg | 96.9 ± 0.5 | 96.9 ± 0.5 | 91.1 ± 1.6 | 91.1 ± 1.6 | 96.4 ± 0.5 | 96.4 ± 0.7 | 94.9 ± 0.7 | 94.9 ± 0.7 |
|  | 15mg | 96.9 ± 0.5 | 96.9 ± 0.5 | 91.1 ± 1.6 | 91.1 ± 1.6 | 96.4 ± 0.5 | 96.4 ± 0.7 | 94.9 ± 0.7 | 94.9 ± 0.7 |

PI: Prevalence of inadequacy: % usual intake below the Physiological or Estimated Average Requirements, SE: standard error (%).

Prevalence of inadequate intake for vitamin A and folate was estimated based on estimated average requirement. Physiological requirements values were applied to estimate the prevalence of inadequate for vitamin B12, zinc and iron from the absorbable nutrient intake. Zinc estimates are based on International Zinc Nutrition Consultative Group (IZiNCG)) physiological requirements values corrected by Hambidge et al. 2011(9). Absorbable vitamin B12, absorbable zinc and absorbable iron were estimated using published algorithms, as described in detail in the text (5, 6, 8).

^1^Nutrient fortification level is expressed per 100 gram of biscuits. A fortification level of 0 indicates total nutrient intakes without added micronutrients in biscuits. RAE: Retinol activity equivalent.

^“^No LSFF” refers to prevalence from natural food sources, in the absence of a large-scale food fortification (LSFF) program. LSFF levels; wheat flour fortification with folic acid (5 mg/kg), vitamin B12 (0.04 mg/kg), zinc (95 mg/kg), iron (60 mg/kg) and edible oil fortification with vitamin A (12 mg/kg).

Effective coverage was calculated by subtracting the prevalence of inadequate intake at each fortification level from the baseline (without added micronutrients in biscuits)

**References**

1. Rice AL, Stoltzfus RJ, de Francisco A, Chakraborty J, Kjolhede CL, Wahed MA. Maternal vitamin A or beta-carotene supplementation in lactating bangladeshi women benefits mothers and infants but does not prevent subclinical deficiency. J Nutr 1999;129(2):356-65. doi: 10.1093/jn/129.2.356.

2. Engle-Stone R, Nankap M, Ndjebayi AO, Vosti SA, Brown KH. Estimating the Effective Coverage of Programs to Control Vitamin A Deficiency and Its Consequences Among Women and Young Children in Cameroon. Food Nutr Bull 2015;36(3 Suppl):S149-71. doi: 10.1177/0379572115595888.

3. WHO. Complementary feeding of young children in developing countries : A review of current scientific knowledge: https://www.who.int/nutrition/publications/infantfeeding/WHO_NUT_98.1/en/. 1998.

4. Miller LV, Hambidge KM, Krebs NF. Zinc Absorption Is Not Related to Dietary Phytate Intake in Infants and Young Children Based on Modeling Combined Data from Multiple Studies. The Journal of Nutrition 2015;145(8):1763-9. doi: 10.3945/jn.115.213074.

5. Armah SM, Carriquiry AL, Reddy MB. Total Iron Bioavailability from the US Diet Is Lower Than the Current Estimate. The Journal of Nutrition 2015;145(11):2617-21. doi: 10.3945/jn.115.210484.

6. Doets EL, In 't Veld PH, Szczecinska A, Dhonukshe-Rutten RA, Cavelaars AE, van 't Veer P, Brzozowska A, de Groot LC. Systematic review on daily vitamin B12 losses and bioavailability for deriving recommendations on vitamin B12 intake with the factorial approach. Ann Nutr Metab 2013;62(4):311-22. doi: 10.1159/000346968.

7. Gibson RS, King JC, Lowe N. A Review of Dietary Zinc Recommendations. Food Nutr Bull 2016;37(4):443-60. doi: 10.1177/0379572116652252.

8. Miller LV, Krebs NF, Hambidge KM. A mathematical model of zinc absorption in humans as a function of dietary zinc and phytate. The Journal of Nutrition 2007;137(1):135-41. doi: 10.1093/jn/137.1.135.

9. Hambidge KM, Miller LV, Krebs NF. Physiological requirements for zinc. International journal for vitamin and nutrition research Internationale Zeitschrift fur Vitamin- und Ernahrungsforschung Journal international de vitaminologie et de nutrition 2011;81(1):72-8. doi: 10.1024/0300-9831/a00052.
